# Carbonic Anhydrase IX in Tumor Tissue and Plasma of Breast Cancer Patients: Reliable Biomarker of Hypoxia and Prognosis

**DOI:** 10.3390/ijms24054325

**Published:** 2023-02-21

**Authors:** Ingeborg Rezuchova, Maria Bartosova, Petra Belvoncikova, Martina Takacova, Miriam Zatovicova, Lenka Jelenska, Lucia Csaderova, Iveta Meciarova, Kamil Pohlodek

**Affiliations:** 1 Institute of Virology, Department of Tumor Biology, Biomedical Research Center of the Slovak Academy of Sciences, Dubravska cesta 9, 845 05 Bratislava, Slovakia; 2 MABPRO, a.s., Dubravska cesta 2, 841 04 Bratislava, Slovakia; 3 Pathology Diagnostic Center, Unilabs Slovakia, 841 01 Bratislava, Slovakia; 4 2nd Department of Gynaecology and Obstetrics, Faculty of Medicine, Comenius University of Bratislava, Ruzinovska 6, 821 01 Bratislava, Slovakia

**Keywords:** carbonic anhydrase IX, breast cancer, hypoxia marker, soluble CA IX, exosomes, prognosis, immunohistochemistry, ELISA

## Abstract

Carbonic anhydrase IX (CA IX) is recognized as an excellent marker of hypoxia and an adverse prognostic factor in solid tumors, including breast cancer (BC). Clinical studies confirm that soluble CA IX (sCA IX), shed into body fluids, predicts the response to some therapeutics. However, CA IX is not included in clinical practice guidelines, possibly due to a lack of validated diagnostic tools. Here, we present two novel diagnostic tools—a monoclonal antibody for CA IX detection by immunohistochemistry and an ELISA kit for the detection of sCA IX in the plasma—validated on a cohort of 100 patients with early BC. We confirm that tissue CA IX positivity (24%) correlates with tumor grading, necrosis, negative hormone receptor status, and the TNBC molecular subtype. We show that antibody IV/18 can specifically detect all subcellular forms of CA IX. Our ELISA test provides 70% sensitivity and 90% specificity. Although we showed that this test could detect exosomes in addition to shed CA IX ectodomain, we could not demonstrate a clear association of sCA IX with prognosis. Our results indicate that the amount of sCA IX depends on subcellular CA IX localization, but more strictly on the molecular composition of individual molecular subtypes of BC, particularly on metalloproteinases inhibitor expression.

## 1. Introduction

Breast cancer (BC) is the most common cause of cancer death in Europe and worldwide, transcending the medical and epidemiological dimension and becoming a serious social and economic problem. An estimated 2.3 million cases of BC in women were diagnosed worldwide in 2020, and approximately 685,000 women died from the disease the same year [1]. Although there is a significant increase in BC cases diagnosed in the early stages of the disease in countries with well-established screening programs, the relative number of deaths from BC is still high. If current trends do not change, the burden of breast cancer is projected to increase to more than 3 million new cases and 1 million deaths per year by 2040 due to population growth and aging alone.

Despite the existence of well-established traditional prognostic markers, such as tumor size, patient age and menopausal status, tumor axillary lymph node involvement, lymphatic/vascular invasion status, hormone receptor status (estrogen (ER) and progesterone (PR)), status of human epidermal growth factor 2 receptor (HER2), and the pathologic prognostic stage (according to the *AJCC Cancer Staging Manual, 8th edition* [2]), clinical practice shows that, solely on the basis of these markers, it is not possible to identify the prognosis of all patients with BC reliably. The 2022 updated ASCO guidelines recommend also using multi-gene tests, such as MammaPrint, Oncotype DX Recurrence Score, Prosigna, EndoPredict, and the Breast Cancer Index [3], to guide certain types of treatment in specific groups of patients. However, there are still populations of BC patients for whom there is no recommendation to use any biomarker. For example, data on the use of genomic testing to guide adjuvant chemotherapy in patients with ≥4 positive nodes are not available. Furthermore, none of these tests are recommended to guide treatment in patients with HER2+ or triple-negative breast cancer (TNBC) [4]. Therefore, the effort to identify new biomarkers that can be investigated by “classical”, cost-effective, easy-to-use and routinely used, easily evaluable, yet sensitive and specific methods, such as immunohistochemistry (IHC) and enzyme-linked immuno-sorbent assay (ELISA), is still relevant.

Carbonic anhydrase IX (CA IX) is a tumor-associated, cell surface glycoprotein, the expression of which is primarily induced by the HIF-1α transcription factor during hypoxia [5]. Basic characteristics of CA IX are: (1) absence in healthy, non-hypoxic, non-tumor tissues (except for some areas of the gastrointestinal tract); (2) overexpression in a wide variety of tumors with the hypoxic phenotype; (3) as a high-activity enzyme catalyzing the reversible conversion of carbon dioxide into bicarbonate ions and protons, is involved in the regulation of intracellular and extracellular pH—it maintains a neutral pH inside tumor cells and contributes to the acidosis of the tumor microenvironment; (4) it facilitates cell migration and invasion with its pH-regulating ability; and (5) its proteoglycan (PG)-like domain participates in tumor cell adhesion and proliferation processes. In summary, by its localization, expression pattern, enzymatic function, and non-catalytic function of the PG-like domain, CA IX promotes tumor cell survival in hypoxia/acidosis and contributes to the increased ability of tumor cells to migrate, invade, and metastasize, as reviewed in [6,7]. Therefore, CA IX is an ideal target that is interesting not only for the design of diagnostic but also therapeutic approaches for the treatment of solid tumors associated with hypoxia.

Comprehensive meta-analysis of clinical studies results has confirmed the significant prognostic importance of IHC diagnostics of CA IX in solid tumors [8]. Patients with high CA IX expression have a higher risk of local failure, disease progression, and a higher risk of metastases developing, independently of tumor type or site. It has been found that the presence of CA IX in tumor tissue may also serve as a predictive marker for radiotherapy and chemotherapy resistance [9,10,11,12,13]. In BC, the presence of CA IX in tumor tissue is significantly associated with impaired overall survival (OS) (HR = 1.90, 95% CI = 1.45–2.50), decreased disease-free survival (DSF) (HR = 1.74, 95% CI 1.34–2.27), worse disease-specific survival (DSS) (HR = 1.75, 95% CI 1.28–2.38), shorter metastasis-free survival (MSF) (HR = 1.76, 95% CI 1.13–2.74), shorter progression-free survival (PFS) (HR = 1.88, 95% CI 1.13 –3.10), and is also associated with a risk of locoregional relapse (HR = 1.37, 95% CI 0.95–1.96) [8].

The cell regulates the presence of CA IX on its surface, similar to HER2, by the cleavage (shedding) of the extracellular portion (ectodomain) of CA IX from the cell surface [14,15,16]. The released CA IX ectodomain (soluble form of CA IX, sCA IX) can be detected in body fluids of cancer patients, by ELISA or by Western blotting. CA IX has also been shown to be a component of exosomes released by tumor cells into the extracellular milieu [17,18].

The presence of sCA IX in serum/plasma has, so far, been investigated in some tumor types, but the data available to date are inconclusive, in part due to the use of incompatible detection platforms [19]. Nevertheless, studies assessing the prognostic significance of sCA IX in the plasma/serum of BC patients clearly showed that patients with elevated sCA IX levels had significantly shorter median PFS and OS [20]. In addition, elevated levels of sCA IX correlate with the presence of visceral metastases and circulating tumor cells [21]. This finding supports experimental evidence of a connection between hypoxia and the release of tumor cells into the bloodstream. Similarly, Brown-Glaberman and colleagues found that sCA IX levels increase significantly in response to anti-angiogenic therapy (paclitaxel + sunitinib) [22]. Recently, Janning and colleagues analyzed the potential of pre-treatment levels of sCA IX to predict the efficacy of bevacizumab in the HER2-negative patients arm of GeparQuinto phase III trial (1160 patients; NCT00567554) that were randomized to receive neoadjuvant chemotherapy alone versus neoadjuvant chemotherapy plus bevacizumab [23]. The pre-treatment sCA IX level helped identify a cohort of patients that are potentially under-treated with neoadjuvant chemotherapy alone and showed that sCA IX is a predictive biomarker for bevacizumab treatment.

Despite many findings on the ability of tissue and serum CA IX to be a prognostic/predictive marker, so far this marker is not routinely used in clinical practice. The extensive (in terms of the number of evaluated patients), long-term (in terms of ability to prognose and predict the success/sufficiency of treatment), and standardized (in terms of the use of standardized and verified diagnostic tools) clinical trials, which would demonstrate the unequivocal suitability of using CA IX as a biomarker and its place in the diagnosis of BC (or other tumors), have not yet been performed. This may also be related to the fact that only a limited number of diagnostic (IVD-certified) kits for the detection of CA IX by IHC and sCA IX in serum/plasma are available on the market, and as we have recently shown, the use of high-quality diagnostic antibodies is critical for the correct interpretation of the results [24]. 

Thus, the aim of this work was to present new diagnostic tools, potentially suitable for IVD certification—an antibody for the detection of CA IX in IHC and an ELISA kit for the detection of sCA IX in plasma—whose diagnostic properties we verified on a cohort of 100 patients with BC. In addition, we also performed a comprehensive in silico analysis of the prognostic utility of CA IX in BC using extensive databases of multi-omics, microarray, RNAseq, and clinical data analyzed by online tools—the Tumor online Prognostic analysis Platform and the Breast Cancer Gene-Expression Miner. 

## 2. Results

### 2.1. Patient Characteristics

One hundred and two patients were enrolled in this single-institutional cohort study between April 2019 and October 2022 at the Breast Unit of the 2nd Department of Gynaecology and Obstetrics, University Hospital of Bratislava, Slovakia. Patients did not undergo radiotherapy or neoadjuvant chemotherapy before surgery and tissue collection. Out of 102 enrolled patients, 100 patients (98.04%) were assessed for CA IX expression in tumor tissue and sCA IX in plasma. Out of the remaining two patients, one patient had a *Phyllodes tumor*, and one patient had a benign breast disease. The clinical and pathologic parameters considered in our study included patient age, grade, stage and size of the tumor, lymph node involvement, tumor necrosis, and the ER, PR, and HER2 receptor status. According to histopathology results, the tumors were categorized into intrinsic subtypes and pathologic prognostic stage groups according to the *AJCC Cancer Staging Manual, 8th edition* [2]. The characteristics of 100 patients included in this study are shown in Table 1.

### 2.2. Immunohistochemical Detection of CA IX in Tumor Tissues

**Figure 1:**
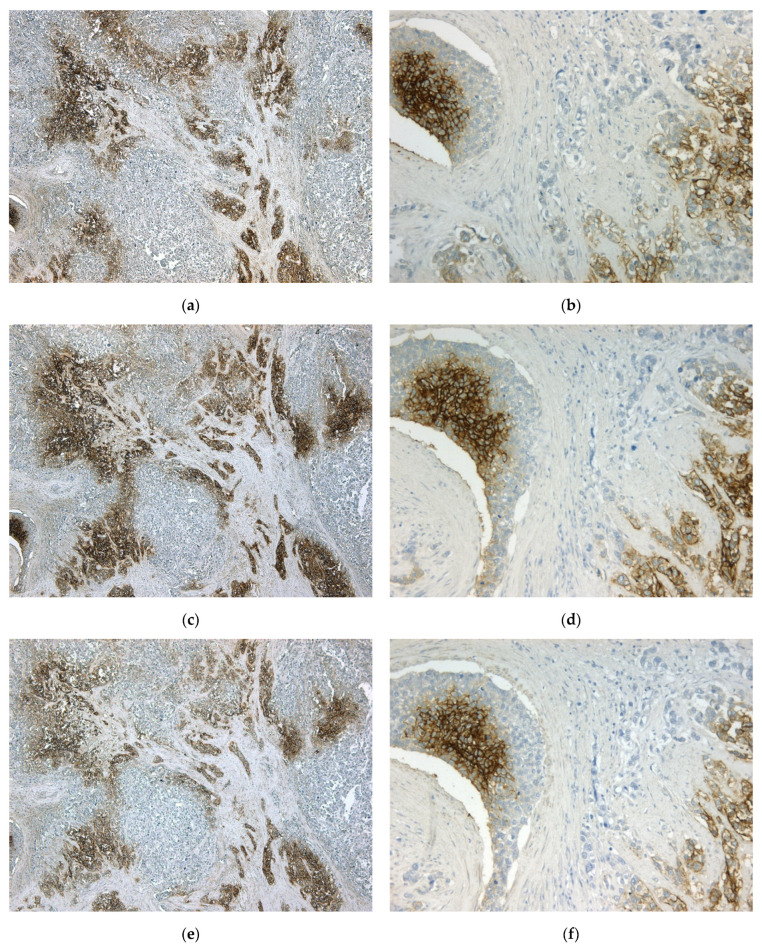

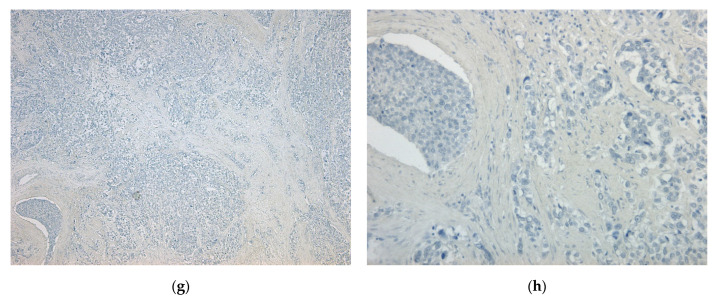
Immunohistochemical staining of TNBC tissue with MAb IV/18 versus M75 versus EP161. (**a**) MAb IV/18 diluted 1:100, magnification ∑ 50×; (**b**) MAb IV/18 diluted 1:100, magnification ∑ 200×; (**c**) M75 diluted 1:1000, magnification ∑ 50x; (**d**) M75 diluted 1:1000, magnification ∑ 200×; (**e**) EP161 diluted 1:100, magnification ∑ 50×; (**f**) EP161 diluted 1:100, magnification ∑ 200×; (**g**) negative control—IHC staining without antibody, magnification ∑ 50×; (**h**) negative control—IHC staining without antibody, magnification ∑ 200×.

**Figure A1:**
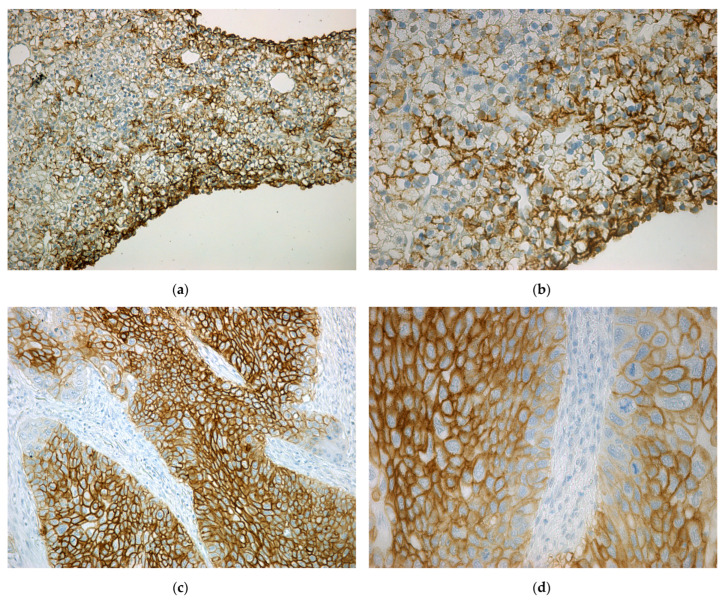

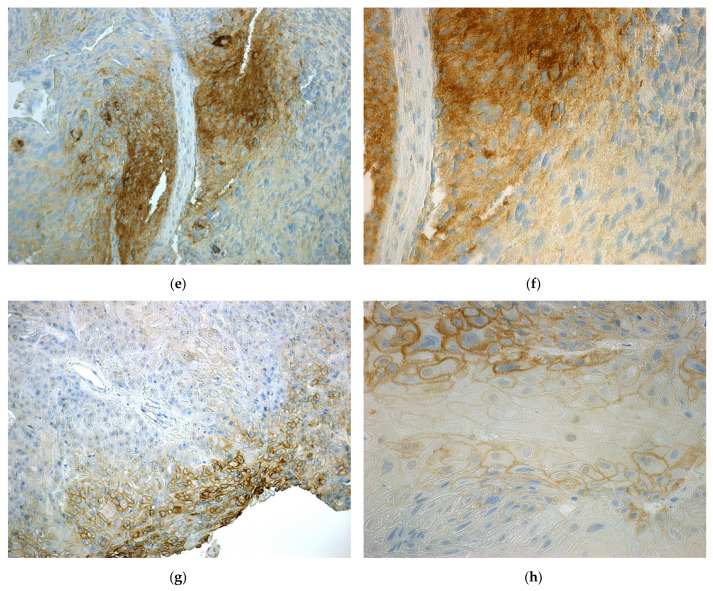
Immunohistochemical staining of human tumor tissues with MAb IV/18. (**a**) Tissue of ccRCC, magnification ∑ 200×; (**b**) tissue of ccRCC, magnification ∑ 400×; (**c**) lung mesothelioma, magnification ∑ 200×; (**d**) lung mesothelioma, magnification ∑ 400×; (**e**) glioblastoma, magnification ∑ 200×; (**f**) glioblastoma, magnification ∑ 400×; (**g**) head and neck carcinoma, magnification ∑ 200×; (**h**) head and neck carcinoma, magnification ∑ 400×.

For IHC detection of CA IX in tumor tissue, we employed a previously unused mouse monoclonal antibody (MAb) IV/18 originating from our earlier prepared collection of anti-CA IX antibodies [25]. MAb IV/18 recognizes a linear epitope on the PG-like domain of human CA IX, which partially overlaps with the epitope of the known M75 antibody [26]. First of all, we evaluated the ability of MAb IV/18 to specifically and sensitively detect CA IX. In IHC staining of TNBC tissue, we compared the staining of CA IX with the MAb IV/18 to the staining provided by the well-established antibodies M75 and the rabbit monoclonal antibody EP161 (Cell Marque^TM^), which is, to the best of our knowledge, the only IVD-certified anti-CA IX antibody available on the European market. The MAb IV/18 provided a strong membrane CA IX staining of comparable intensity and extent to antibodies M75 and EP161 (Figure 1). The IHC staining of other tumor type tissues with MAb IV/18 can be seen in Appendix A, Figure A1.

In our cohort of 100 patients with BC, we detected a total of 24 CA IX-positive (CA IX+) cases (24.00%) (Table 2). We considered cell membrane CA IX staining, mixed staining of membrane and cytoplasm, and cytoplasmic staining (33.33% of CA IX+ cases) as positive. The presence of CA IX in the tissue was not detected in 76 cases (76.00%). We also recorded 18 cases (18.00%) of CA IX nuclear staining, which we classified as negative. 

In statistical analysis comparing clinicopathological variables with CA IX expression (Table 2), the tissue CA IX (tCA IX) positivity significantly correlated with tumor grading (*p = 0.0146*), necrosis (*p = 0.0015*), the ER status (*p = 0.0019*), and with molecular subtypes of BC (*p = 0.0115*). The tCA IX was positive in 52.38% of tumors with necrosis, in 38.89% of grade III tumors, in 34.15% of large tumors (pT2-4) (non-significant (*ns*)), and in 27.78% of those with 1–3 nodes affected (pN1-3) (*ns*). 

The tCA IX positivity was associated with the ER- (50.00% cases; *p = 0.0019*) and PR- (36.84% cases; *ns*) receptor status. The only parameter in which our IHC analysis of CA IX expression in BC does not correspond with previously published data [27,28] is tCA IX positivity versus HER2 receptor status. In our case, tCA IX-positive (tCA IX+) were 25.00% of HER2- tumors, while only 18.75% of HER2+. However, it should be noted that, overall, we recorded a lower number of HER2+ cases in our cohort (only 16.00%). The most significant was the association of tCA IX+ expression with TNBC (up to 66.67% of these cases were positive). This was followed by the luminal B-like molecular subtype (27.78%), HER2+ (18.75%), and luminal A (16.67%). 

A CA IX score was established to qualitatively and quantitatively assess tissue CA IX positivity. Based on the subcellular localization of CA IX, the relative number of positively stained cells, and the staining intensity, CA IX-positive cases were divided into three categories: low, focal, and high expression of tCA IX (see Materials and Methods). As shown in Table 2, high tCA IX positivity was significantly associated with high tumor grade (*p = 0.0060*) and the presence of necrosis (*p = 0.0062*). Although statistical analysis did not confirm significance, the data show that high tCA IX also occurred in TNBC and luminal B-like molecular subtypes, in large tumors (pT2–4), and in tumors with ER/PR- status. 

As already mentioned, we also observed 18 cases of nuclear CA IX staining, which we did not consider as tCA IX+. However, CA IX nuclear staining in luminal A and HER2 molecular subtypes, in cases with lower grading, positive necrosis, and ER- status, was evaluated as significant (Appendix B, Table A1).

### 2.3. Evaluation of Soluble CA IX in Patient Plasma 

**Figure 2:**
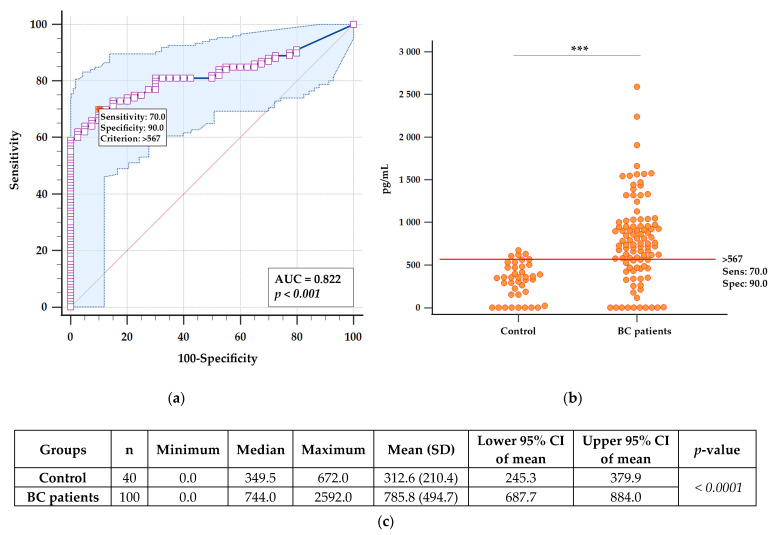
Receiver operating characteristic (ROC) curve analysis of CA IX ELISA: (**a**) ROC curve analysis was performed by MedCalc^®^ Statistical Software version 20.2 (MedCalc Software Ltd., Ostend, Belgium; https://www.medcalc.org (accessed on 1 October 2022); 2022) based on sCA IX values of BC patients included in the study versus healthy volunteers (n = 40). The software calculated the area under the curve (AUC), the statistical significance of the difference between the groups of healthy (Control) and tested BC patients (*p < 0.001*), and determined the best cut off according to Youden’s statistic; (**b**) interactive dot diagram of measured levels of sCA IX in both groups with the cut off line (red horizontal line). The statistical significance of the difference between the groups of healthy (Control) and tested BC patients was evaluated by Mann–Whitney test for independent samples (*** Indicates significant effects with *p < 0.0001*); (**c**) the table shows summary statistical data of sCA IX (pg/mL) for each group: number of patients (n), minimum, median, maximum, mean with standard deviation (SD), lower 95% confidence interval (CI) for mean, upper 95% CI for median, and result of Mann–Whitney test for independent samples (*p < 0.0001*).

To determine the level of soluble CA IX in the preoperative plasma of BC patients, we used an ELISA kit developed earlier [29], with minor modifications (see Materials and Methods). To determine whether sCA IX is related to the disease, we compared the sCA IX plasma level of BC patients with that of apparently healthy volunteers (n = 40) and identified the optimal cut off based on Youden’s criterion maximizing Youden’s J statistic (sensitivity + specificity). As shown by a receiver operating characteristic (ROC) curve in Figure 2A (AUC= 0.822: 95% CI: [0.4601 to 0.6700]), using this test with a cut off of 567 pg/mL, according to the Youden’s statistic, can yield sensitivity of 70% and specificity of 90%. The difference between the compared groups (Control versus BC patients) was statistically significant *p < 0.001*. 

**Figure 3:**
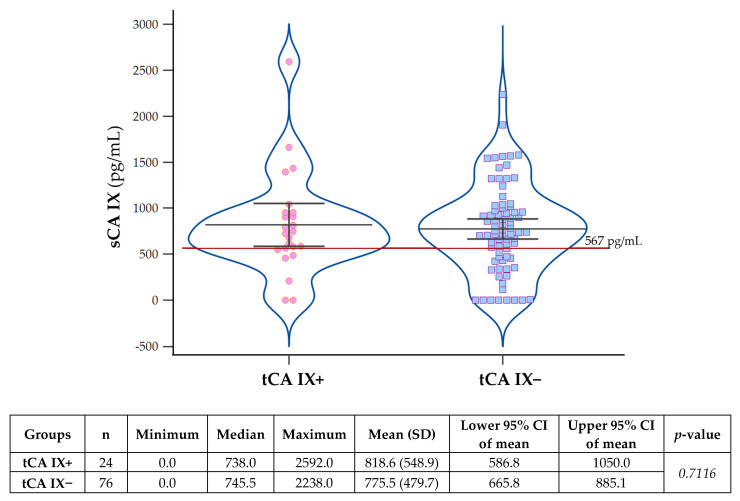
Analysis of sCA IX levels in plasma of patients with CA IX-positive versus CA IX-negative tissue. Violin graphs with dots (plot all data) showing the distribution of sCA IX levels (pg/mL) in tCA IX+ and tCA IX- groups determined by IHC. The table shows summary statistical data of sCA IX (pg/mL) for each group: number of patients (n), minimum, median, maximum, mean with standard deviation (SD), lower 95% confidence interval (CI) for mean, upper 95% CI for median, and result of unpaired *t*-test (*p* = *0.7116*).

We confronted sCA IX plasma levels with CA IX positivity in tumor tissue and found that patients with tCA IX+ did not have significantly higher levels of sCA IX compared to those with tCA IX- (Figure 3). The average concentration of sCA IX in patients with tCA IX+ was 818.6 pg/mL, while in patients with tCA IX- it was 775.5 pg/mL. Of the 24 patients with tCA IX+, 17 had sCA IX higher than the cut off value. The remaining seven had sCA IX below the cut off value, and in two of them we did not detect the presence of sCA IX at all (details about individual patients can see in Table 3, below). Out of 76 patients with tCA IX-, up to 53 patients had sCA IX above the cut off value. The analysis of association between levels of sCA IX above the cut off and below the cut off value with tCA IX+ and tCA IX- groups did not show statistical significance according to Fisher’s exact test (*p = 0.5967*). 

**Figure A2:**
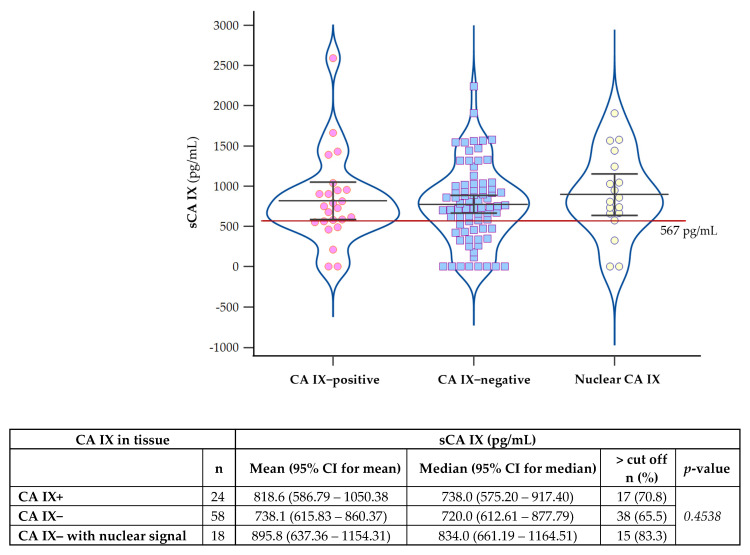
Analysis of sCA IX levels in plasma of patients with CA IX nuclear positivity in tumor tissue. Violin graphs with dots (plot all data) showing the levels of sCA IX in groups CA IX−positive versus CA IX−negative versus CA IX−negative with nuclear signal (determined by IHC). Table displaying summary statistical data for each group: number of patients (n), mean with 95% confidence interval (CI) for mean, median with 95% CI for median, number of patients with sCA IX level above the cut off, and *p*-value (Kruskal–Wallis test with Dunn’s post hoc test).

When we selected from the group of tCA IX- patients, those in whom we noted nuclear staining of CA IX in the tissue, we found that they had slightly higher levels of sCA IX (Appendix B, Figure A2). Even in this case, statistical analysis did not show this difference to be significant (*p = 0.4538*). 

**Figure 4:**
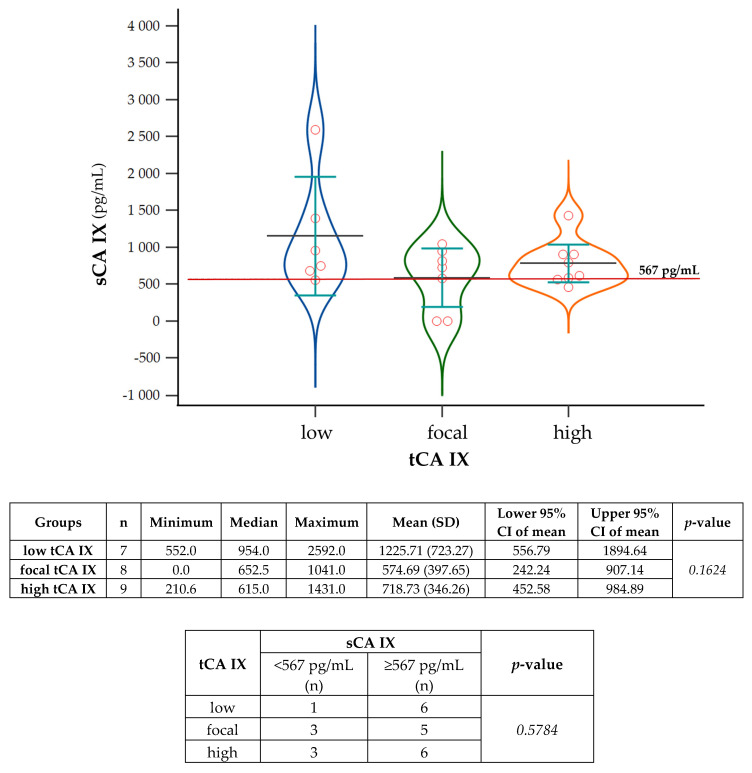
Analysis of sCA IX levels in the plasma of patients with CA IX+ tumor tissue. Violin graphs with dots (plot all data) showing sCA IX levels of patients divided according to CA IX score into groups: low, focal, and high tCA IX. For the evaluation of differences in sCA IX levels between tCA IX groups, the Kruskal–Wallis test with Dunn’s post hoc test was used (*p* = *0.1624)*. Summary statistical data of sCA IX levels (n= number of patients, minimum, median, maximum, mean with standard deviation (SD), lower and upper 95% CI of mean, and *p*-value) for each group are displayed in the table below the image. The lower table expresses the association between sCA IX below (<567 pg/mL) and above (≥567 pg/mL) the cut off value with the low, focal, and high tCA IX groups. The statistical evaluation was performed using a chi-squared test. (n)—number of patients.

The analysis of sCA IX levels in patients, divided according to CA IX score into low, focal, and high tCA IX+ groups, revealed that patients with low tCA IX had the highest plasma sCA IX levels (Figure 4). Except for one patient (however, with a sCA IX value close to the cut off), all had sCA IX levels above the cut off value (details about individual patients can see in Table 3). The average concentration of sCA IX in the plasma of this group was 1225.71 pg/mL. Similarly, most patients (six of nine) from the high tCA IX group had sCA IX levels higher than the cut off value (mean = 718.73 pg/mL). In contrast, in the third group, patients with focal tCA IX, had plasma sCA IX levels close to the cut off value, the mean was only 574.69 pg/mL (Figure 4). However, statistical analysis did not confirm the significance of these differences (*p = 0.1624*).

In Table 3, we present the clinicopathological characteristics of patients with tCA IX+ and levels of sCA IX they had in their plasma. Although the majority of tCA IX+ patients had sCA IX levels above the cut off value, we did not confirm the statistical significance of this fact. Therefore, we can conclude that in our patient’s cohort we did not find a direct association between the amount of CA IX expressed in tumor tissue and the level of sCA IX in their plasma. Even considering the high levels of sCA IX in patients without tCA IX+, we assume that the importance of sCA IX will probably lie more in the prediction of treatment response, as already found by other authors [22,23], than in prognosis. As the patients included in our study are still on treatment or very soon after treatment, we cannot yet evaluate the predictive value of sCA IX. 

Since, CA IX has also been shown to be a component of exosomes released by tumor cells into the extracellular milieu [17,18]. We, therefore, wondered if we were able to detect exosomal CA IX with our ELISA. As documented in Appendix C, our ELISA test is also able to detect CA IX, which is a component of exosomes. Interestingly, we found that the patient with a very high sCA IX plasma (1440.00 pg/mL), who at the same time was tCA IX-negative with nuclear CA IX signal, had the greatest number of CA IX-positive exosomes. However, it should be noted that the test was performed on a sample of only four patients. Therefore, we cannot reliably say whether there is a direct association between tCA IX positivity or tCA IX subcellular localization and the number of CA IX-positive exosomes, and we also do not know the proportion of CA IX-positive exosomes to total sCA IX. 

### 2.4. In Silico Analysis of CA IX Expression in Breast Cancer 

**Figure 5:**
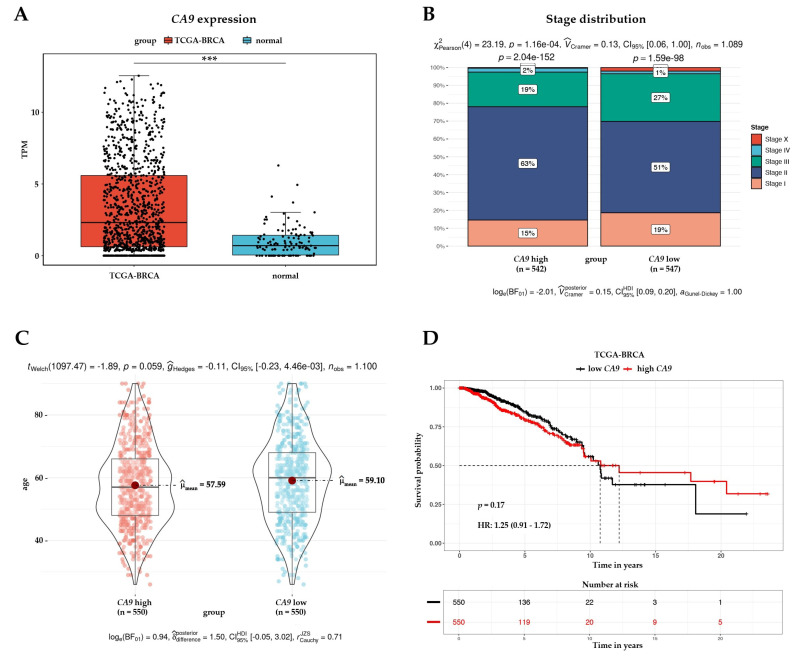
Analysis of *CA9* gene expression in the TCGA-BRCA dataset. (**A**) Differential expression between tumor and normal tissue presented as a boxplot for the two groups. The Wilcox test was performed to test whether there was a significant difference between the two groups. *** *p* < 0.001. Stage distribution (**B**) and age at initial pathologic diagnosis (**C**) are presented based on the expression status of the *CA9* gene; (**D**) univariate analysis presented as a Kaplan–Meier (KM) survival plot includes the *p*-value (*p = 0.17*) and hazard ratio with 95% confidence interval information.

In order to explore the expression as well as the prognostic value of CA IX in BC, we performed a comprehensive in silico analysis using the Tumor online Prognostic analysis Platform (ToPP) [30] and the Breast Cancer Gene-Expression Miner [31]. Figure 5 summarizes data from the ToPP, which were acquired after the selection of The Cancer Genome Atlas–Breast Invasive Carcinoma (TCGA-BRCA) dataset. A univariate analysis of CA IX revealed a significantly elevated expression of the *CA9* gene in tumor tissue (Figure 5A). Based on the expression status of *CA9* (*CA9* high/low), stage distribution as well as age at initial pathologic diagnosis are depicted in Figure 5B,C, respectively. Although not statistically significant, the OS according to the *CA9* expression status is presented in Figure 5D. 

**Figure 6:**
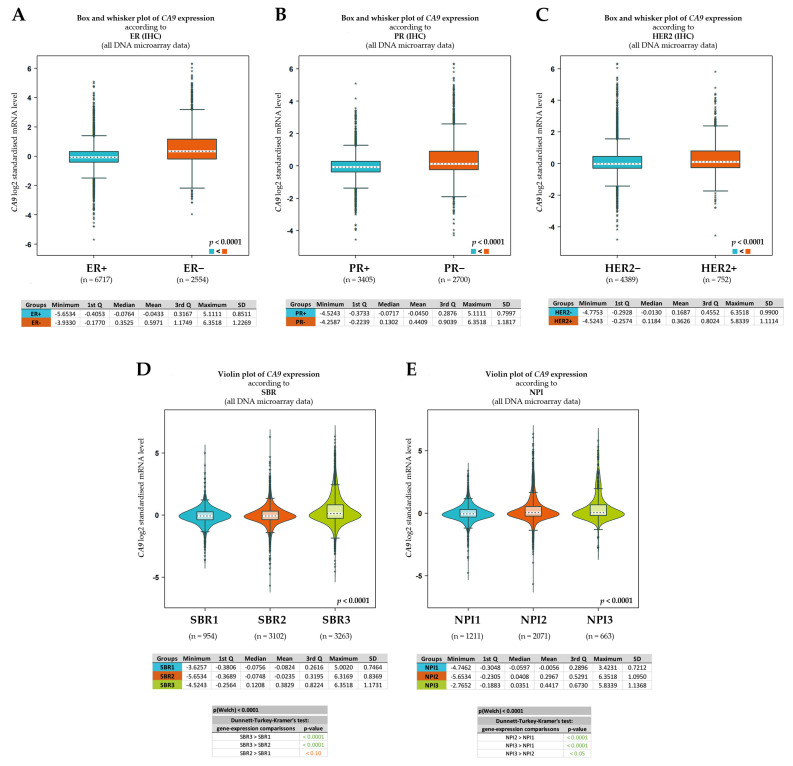
*CA9* gene expression analysis according to receptor (ER, PR, and HER2) statuses, Scarff–Bloom–Richardson grade, and Nottingham prognostic index. Expression analysis for *CA9* with positive versus negative receptor IHC status: ER (**A**); PR (**B**); and HER2 (**C**) (n = 9335); (**D**) expression analysis for *CA9* with 1 versus 2 versus 3 Scarff–Bloom–Richardson (SBR) grade (n = 7319); (**E**) expression analysis for *CA9* with 1 versus 2 versus 3 Nottingham prognostic index (NPI) status (n = 3945). Results are displayed as box and whiskers (**A**–**C**) and violin graphs (**D**,**E**) showing the distribution of *CA9* expression according to groups. Significant differences between groups are assessed by Welch’s test, and the corresponding *p*-value is indicated on the bottom right of the figure. In case of more than two groups, a Dunnett–Tukey–Kramer test is presented for each pairwise comparison. Summary statistical data (minimum, first quartile, median, mean, third quartile, maximum, and standard deviation) for each group are displayed in tables.

To shed more light on the prognostic significance of CA IX in BC, we examined *CA9* gene expression according to various clinicopathological characteristics using the Breast Cancer Gene-Expression Miner. Target gene expression analysis enabled us to reveal significantly different *CA9* expression between two groups of patients, ER+ and ER- (Figure 6A). In addition to the ER status, *CA9* expression was examined according to the PR and HER2 status. Similarly, as in the case of the ER- group, a significantly upregulated expression of *CA9* mRNA was confirmed in the PR- (Figure 6B) and in the HER2+ (Figure 6C) groups. 

Furthermore, an increased Scarff–Bloom–Richardson (SBR) grade was correlated with the increased level of the *CA9* transcript (Figure 6D). The SBR grade, an important prognostic factor in BC, is associated with cell proliferation. Amat and colleagues demonstrated the predictive significance of the SBR grade both on clinical and pathological responses to chemotherapy [32]. As shown in Figure 6D, a significant difference in *CA9* mRNA levels was revealed when the highest grade (SBR3) was compared to SBR1 and SBR2. No statistical difference in *CA9* expression was observed between SBR1 and SBR2. In addition to the SBR grade, another established prognostic indicator, the Nottingham prognostic index (NPI), was examined based on the expression status of *CA9*. The NPI takes into account the size of the tumor, number of lymph nodes involved, and tumor grade, and its validity has been reviewed [33,34]. As shown in Figure 6E, a significant difference in *CA9* expression was observed in all three comparisons.

## 3. Discussion

Carbonic anhydrase IX is undeniably an excellent endogenous marker of tumor hypoxia and its high expression in tumor tissue is clearly an adverse prognostic marker, as has been extensively reviewed [6,7,8]. Furthermore, our comprehensive in silico analysis, performed on the annotated breast cancer transcriptomic data (n = 9335), confirmed that the presence of CA IX in breast tumor tissue significantly correlates with ER-, PR-, and HER2+ statuses, and with the highest grade of SBR and NPI (Figure 5 and Figure 6). However, CA IX is not used in clinical practice despite clear evidence of its usefulness as a biomarker. There may be several reasons why. Probably the most serious reason is the lack of reliable and validated diagnostic antibodies and kits, and a related reason is the absence of a prospective trial(s) with a randomized patient cohort that would provide high-quality evidence for CA IX prognostic validation.

The main goal of our study was to introduce new diagnostic tools, potentially suitable for IVD certification—an antibody for the detection of CA IX in IHC and an ELISA kit for the detection of sCA IX in the plasma—and to verify their diagnostic properties on a cohort of 100 patients with primary operable early BC. For this purpose, we decided to use the MAb IV/18, originating from our collection of CA IX-specific antibodies [25], and an ELISA kit developed earlier [29]. First of all, we verified the ability of MAb IV/18 to specifically and sensitively detect CA IX in FFPE specimens by IHC staining. The MAb IV/18 was able to provide strong and specific staining of CA IX on membranes of TNBC cells, comparable to M75 and EP161 antibodies (Figure 1). In our cohort of BC patients, we diagnosed 24% of CA IX-positive cases by IHC (Table 2). We detected tCA IX in 52.38% of tumors with necrosis (*p = 0.0015*), in 38.89% of grade III tumors (*p = 0.0146*), in 34.15% of large tumors (pT2-4), and in 27.78% of those with 1–3 nodes affected. Moreover, high tCA IX positivity (according to CA IX score) was also significantly associated with high tumor grade and the presence of necrosis. We detected a significant association of tCA IX positivity with negative hormone receptor status (*p = 0.0019*). CA IX tissue positivity was also related to BC molecular subtypes (*p = 0.0115*), especially TNBC (up to 66.67% of these cases were tCA IX+), then luminal B-like (27.78%), HER2+ (18.75%), and luminal A (16.67%) subtype. In our patient cohort, we confirmed that tissue CA IX is a good biomarker that usefully complements currently used prognostic markers in early BC.

**Figure A6:**
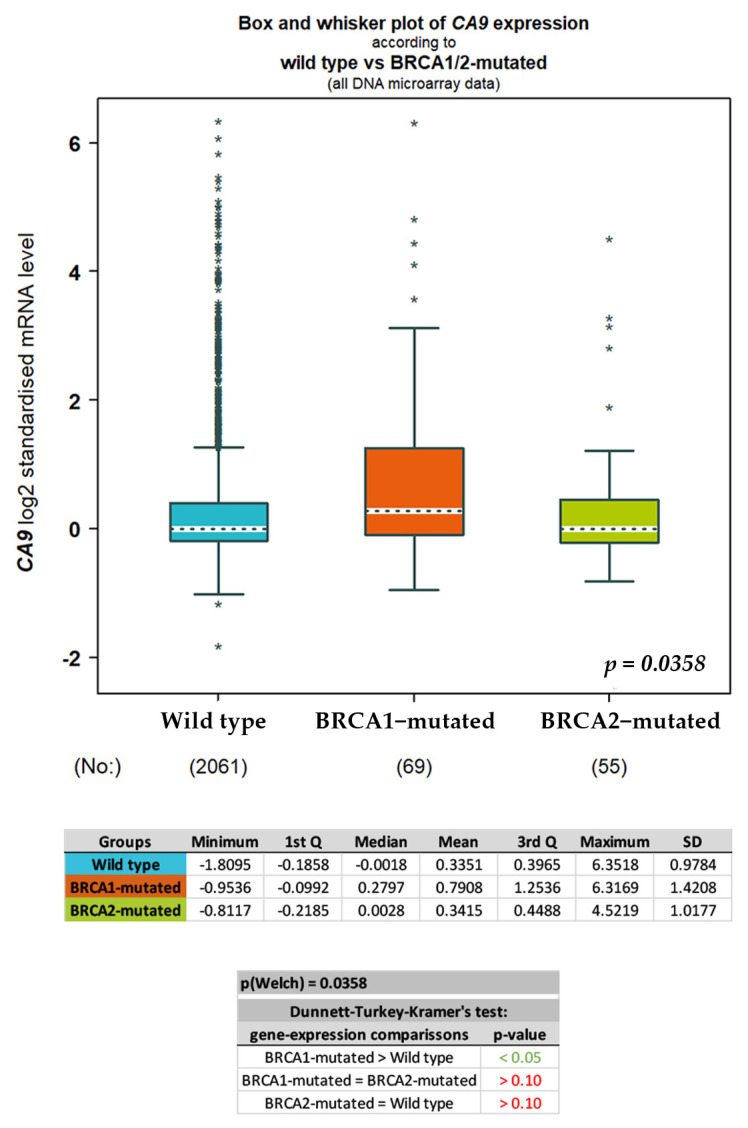
Analysis of *CA9* gene expression according to wild type versus mutated BRCA 1/2 genes. Expression analysis for *CA9* gene with wild type BRCA 1/2 (n = 2061) versus BRCA1-mutated (n = 69) versus BRCA2-mutated (n = 55) gene. Results are displayed as box and whiskers graphs showing the distribution of *CA9* expression according to groups. Significant differences between groups are assessed by Welch’s test, and the corresponding *p*-value is indicated on the bottom right of the figure. For each pairwise comparison, a Dunnett–Tukey–Kramer’s test was used and is presented in the upper table. Summary statistical data (minimum, first quartile, median, mean, third quartile, maximum, and standard deviation) for each group are displayed in the bottom table. Analysis was performed using the Breast Cancer Gene-Expression Miner v4.9 software.

The only parameter in which our IHC analysis of CA IX expression in BC does not fully correspond with in silico analysis data (Figure 6) and earlier observations [27,28] is the significant association of CA IX positivity with HER2+ receptor status. In our study, up to 25.00% of HER2- tumors were tCA IX+, while only 18.75% of HER2+ tumors were tCA IX+. However, it should be noted that, overall, we recorded a lower number of HER2+ cases in our cohort (only 16.00%). Nevertheless, our results on the frequency of CA IX expression in BC tissue and its association with clinicopathological characteristics correspond with the findings of other authors [8,9,35,36,37]. CA IX expression in BC tissue is also known to be associated with the BRCA1 mutation [38,39], which we also document as a result of our in silico analysis in Figure A6 (Appendix D). Since no patient in our cohort was suspected of having hereditary BC, we did not investigate BRCA status, and therefore, we could not verify this fact. 

To determine the level of soluble CA IX in the preoperative plasma of BC patients, we used an ELISA kit developed earlier [29] with minor modifications (see Materials and Methods). We compared the sCA IX plasma levels of BC patients with that of healthy volunteers (n = 40) and identified the optimal cut off based on Youden’s criterion maximizing Youden’s J statistic (Figure 2A). With this ELISA setup, using a cut off of 567 pg/mL, we achieved 70% sensitivity and 90% specificity. In the control group, which consisted of apparently healthy volunteers without cancer disease, we detected sCA IX in the range of 0.0–672.0 pg/mL (median = 349.5 pg/mL). In contrast, in the group of patients with BC, we detected sCA IX in the range of 0.0–2592.0 pg/mL, with a median of 744.0 pg/mL. The difference between the control group and the group of BC patients was statistically significant *p < 0.001* (Figure 2B,C). 

Considering the cut off value of 567 pg/mL, a total of up to 70 patients had sCA IX in their plasma above this value, of which 17 were CA IX+ in tissue. However, patients with tCA IX+ did not have significantly higher levels of sCA IX compared to those with tCA IX- (Figure 3). The analysis of sCA IX levels in tCA IX+ patients, divided by CA IX score into low, focal, and high groups, revealed that patients with low tCA IX had higher sCA IX levels (mean = 1225.71 pg/mL) in their plasma (albeit not significantly) than those with focal (mean = 574.69 pg/mL) and high tCA IX (mean = 718.73 pg/mL) (Figure 4). We found that seven patients with tCA IX+ (29.2%) had sCA IX below the cut off value (Table 3). 

In the group of patients with tCA IX-, we detected sCA IX in their plasma in the range of 0.0 to 2238.0 pg/mL (mean = 775.5 pg/mL) (Figure 3). Interestingly, when we selected those who had nuclear staining of CA IX in the tissue from this group of CA IX- patients, we found that they had slightly higher levels of sCA IX in their plasma (mean = 895.8 pg/mL) than those who had no CA IX in their tissue (mean = 738.1 pg/mL) (Figure A2, Appendix B). Our results show that there is not always a relation between the amount of CA IX in tissue and sCA IX in plasma. 

**Figure A7:**
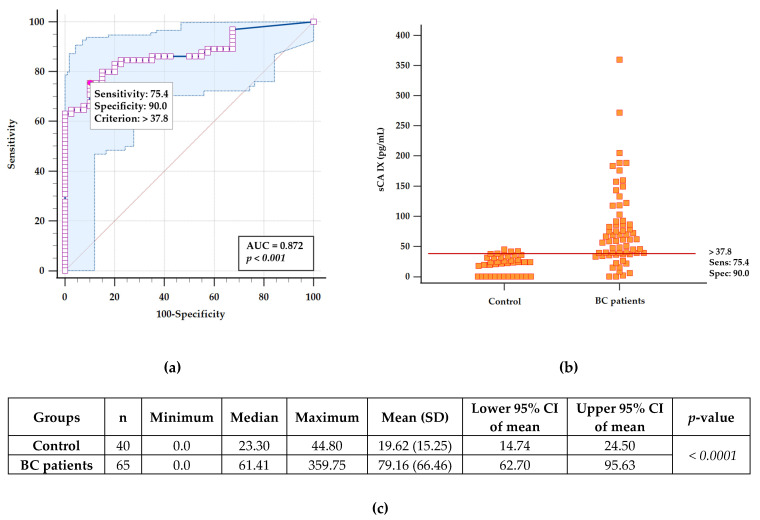
Receiver operating characteristic (ROC) curve analysis of Quantikine^®^ Human Carbonic Anhydrase IX Immunoassay (R&D ELISA): (**a**) ROC curve analysis was performed by MedCalc^®^ Statistical Software version 20.2 based on sCA IX values of BC patients (n = 65) included in the study versus apparently healthy volunteers (n = 40). The software calculated the area under the curve (AUC), the statistical significance of the difference between the groups of healthy (Control) and tested BC patients (*p < 0.001*), and determined the best cut off according to Youden’s statistic; (**b**) interactive dot diagram of measured levels of sCA IX in both groups with the cut off line (red horizontal line); (**c**) the table shows summary statistical data of sCA IX (pg/mL) for each group: number of patients (n), minimum, median, maximum, mean with standard deviation (SD), lower 95% confidence interval (CI) for mean, upper 95% CI for median, and result of the Mann–Whitney test for independent samples (*p < 0.0001*).

**Figure A8:**
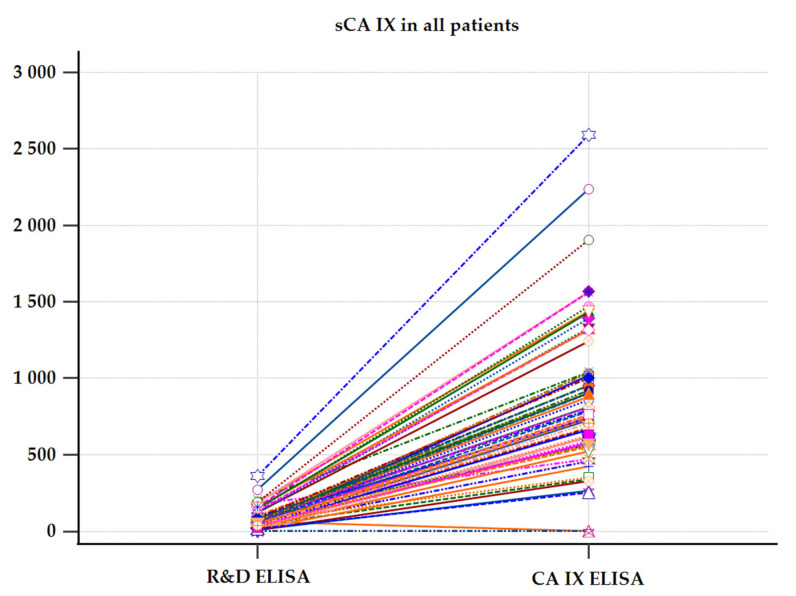
Comparison of R&D ELISA test versus CA IX ELISA. Dot and line diagram of pairwise comparison shows sCA IX concentrations of individual patients (each patient marked with an individual point symbol) measured by R&D ELISA (left) versus CA IX ELISA (right).

**Figure A9:**
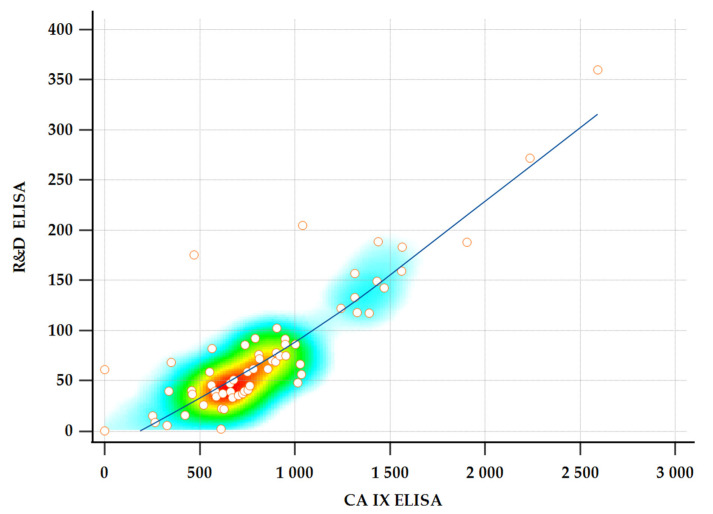
Scatter diagram of the degree of correlation between sCA IX concentrations measured by R&D ELISA versus CA IX ELISA.

**Figure A10:**
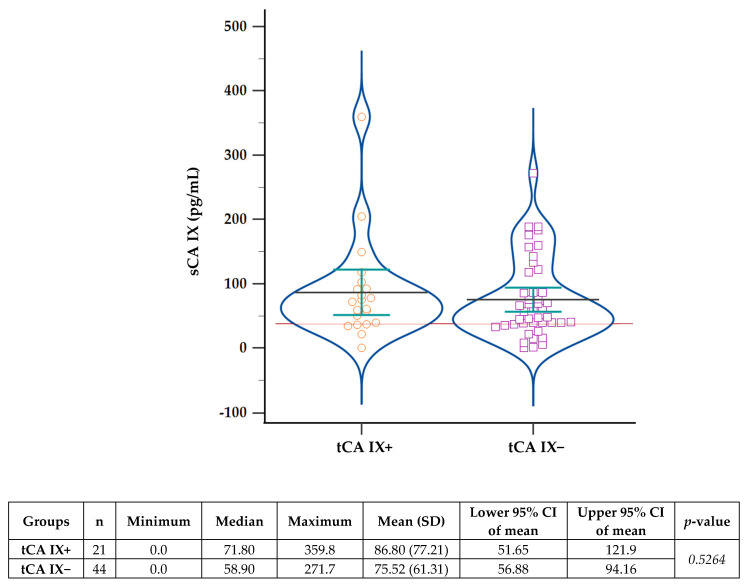
Analysis of sCA IX levels in plasma patients with CA IX-positive versus CA IX-negative tissue using R&D ELISA. Violin graphs with dots (plot all data) showing the distribution of sCA IX levels in tCA IX+ and tCA IX- groups determined by IHC. Cut off value, 37.8 pg/mL, is shown as red horizontal line. The table shows summary statistical data of sCA IX (pg/mL) for each group: number of patients (n), minimum, median, maximum, mean with standard deviation (SD), lower 95% confidence interval (CI) for mean, upper 95% CI for median, and the result of the unpaired *t*-test (*p* = *0.5264*).

**Figure A11:**
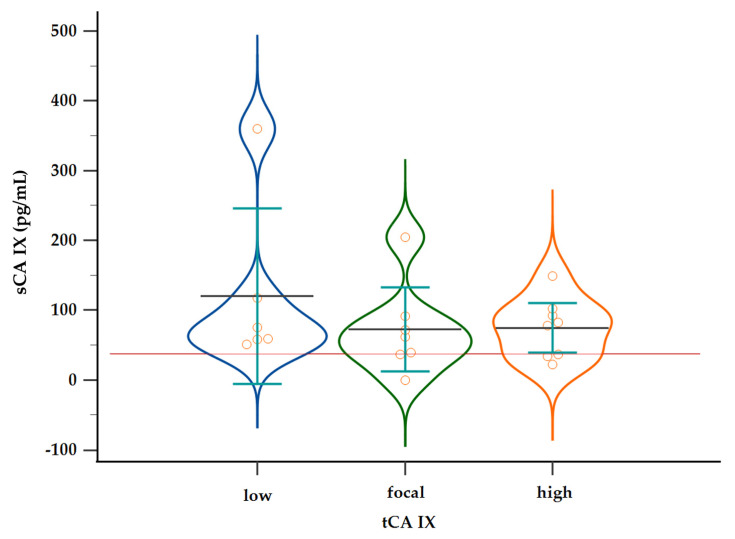
Analysis of sCA IX levels in the plasma of patients with the CA IX+ tumor tissue using R&D ELISA. Violin graphs with dots (plot all data) showing sCA IX levels of patients divided based on CA IX score into groups: low, focal, and high tCA IX. Cut off value, 37.8 pg/mL, is shown as red horizontal line. Differences between groups were assessed by Kruskal–Wallis test with Dunn’s post hoc test with the result *p* = *0.7221*.

Therefore, to verify our measurements, we examined sCA IX of some of our patients (n = 65) and the entire control group (n = 40) using a commonly used commercial kit (intended for research use only (RUO))—Quantikine^®^ Human Carbonic Anhydrase IX Immunoassay (R&D Systems; DCA900) (Appendix E). The ROC curve (AUC = 0.872: 95% CI: [0.5115 to 0.7365]) shows that using a Youden statistic cut off of 37.8 pg/mL, this test can provide sensitivity of 75.4% and specificity of 90% (Figure A7). The sensitivity of this test was only slightly higher (75.4%) than ours (70%); the specificity was the same (90%). In the dot and line plot of the pairwise comparison (Figure A8), as well as in the scatter plot of the degree of correlation (Figure A9), we showed that the sCA IX concentrations measured by both our CA IX ELISA kit and the R&D ELISA are correlated. Similar to our ELISA, using the R&D ELISA we found that tCA IX+ patients did not have significantly higher sCA IX levels compared to tCA IX- patients (Figure A10). The average concentration of sCA IX in patients with tCA IX+ was 86.8 pg/mL, while in patients with tCA IX- it was 75.52 pg/mL. Of the 21 patients with tCA IX+, 16 had sCA IX higher than the cut off value. These were the same patients who had sCA IX above the cut off value when examined by our ELISA. The remaining five had sCA IX below the cut off value, and in one of them we did not detect the presence of sCA IX at all. Out of 44 patients with tCA IX-, up to 33 patients had sCA IX above the cut off value. Thus, the differences between sCA IX levels in the tCA IX+ and tCA IX- groups were not statistically significant (*p = 0.5613*). Similarly, statistical analysis did not confirm significant differences between sCA IX levels in patients with low, focal, and high tCA IX+ (*p = 0.7221*) (Figure A11). Equally to our ELISA, we also found sCA IX levels below the cut off value in patients with tissue CA IX positivity with the R&D kit, and conversely, sCA IX levels above the cut off value occurred in patients without CA IX in the tissue. Therefore, the fact that we cannot clearly distinguish patients with tumor-associated CA IX from patients without CA IX in tumor tissue using plasma sCA IX levels is unrelated to the detection properties of our ELISA.

There are several possible explanations why there is not always a relationship between the amount of CA IX in tissue and sCA IX in plasma. First of all, it is necessary to look at the ectopic expression of CA IX in tumor tissue, as well as the subcellular localization of CA IX (which seem to be related). Immunohistochemical analysis of CA IX expression in tumor tissues revealed that CA IX is present predominantly in tumor areas that actually suffer from hypoxia, but also in areas that are already reoxygenated (CA IX protein is stable in reoxygenated cells for around 38 h) [40]. The pattern of CA IX expression in tumor tissue was described as a continuum without a clear boundary between oxygenated and hypoxic cells and gradually increases towards necrotic areas [41]. Under severe hypoxic conditions, the full-length CA IX protein is expressed and localized on the cell membrane, where, as a part of the bicarbonate transport metabolon, it fulfills its pH regulatory function by catalyzing the conversion of CO_2_ to bicarbonate ions, as has been reviewed [6,7]. However, it has been shown that tumor cells located at shorter distances from functional blood vessels are exposed to mild hypoxia and can express comparable levels of both the full-length CA IX and also an alternatively spliced variant of CA IX [42]. The alternatively spliced variant of human *CA9* mRNA results from the deletion of exons 8 plus 9 and is translated into a truncated protein that lacks the transmembrane region, the intracellular terminus, and the *C*-terminal part of the catalytic domain. It is the removal of the transmembrane and intracellular regions that is apparently responsible for the cytoplasmic localization of this CA IX protein variant. In addition to being differently localized, the alternatively spliced variant of CA IX is differently regulated (also independently of hypoxia) and functionally reduced compared to full-length CA IX. It should be noted that this CA IX protein variant can be released into the extracellular space [42] and potentially detected by our ELISA kit.

Another potential source of cytoplasmic CA IX staining in tissue may be CA IX protein internalized from the cell surface to the cell interior via endocytosis. Endocytosis can be induced by physiological stress, including hypoxia and calcium depletion, but also by specific antibodies against CA IX that bind to its extracellular domain [43,44,45]. For example, one such antibody is MAb VII/20 (created by our group), which targets the catalytic domain of CA IX and can trigger antibody-mediated endocytosis, leading to the depletion of approximately 30% of CA IX molecules from the cell surface within 3 h. Intracellular antibody signal overlapping with the signal of CA IX protein can be detected even during the following 24 h. Then, the complex of antibody-CA IX is recycled back to the cell membrane and CA IX is exposed again on the cell surface [46].

CA IX is thought to undergo endocytosis via clathrin-coated vesicles, early endosomes, in the cytoplasm. Next, lipids, genes, and proteins (including CA IX) are sorted into early endosomes to form numerous intraluminal vesicles that mature into multivesicular bodies. The fate of multivesicular bodies is to fuse with lysosomes and degrade. However, certain multivesicular bodies are released in the extracellular vicinity as exosomes by exocytic fusion with the cell membrane, and some go instead to perinuclear recycling vesicles [46,47]. Although experimental evidence is not available, it is quite possible that a certain fraction of endocytosed CA IX molecules also reaches the cell nucleus. Buanne and colleagues provided the first evidence that CA IX interacts with proteins involved in nuclear/cytoplasmic transport, gene transcription, and protein stability [48]. They identified the nucleo-cytoplasmic trafficking machinery as a distinctive feature of the CA IX interactome, and in this case, nuclear CA IX positivity is not associated with necrotic/inflammatory areas, but rather with cancer tissue regions containing tightly linked neoplastic cells and a limited fibrovascular network. These data showed that there is a nuclear CA IX protein subpopulation with a potential intracellular function, distinct from the crucial CA IX role at the cell surface. 

All of these processes associated with endocytosis can lead to depletion of CA IX from the surface of tumor cells, but, on the other hand, generate potential messengers of autocrine and/or paracrine signaling. These aspects of CA IX regulation clearly require further investigation. Nevertheless, they at least partially explain why membrane, membrane/cytoplasmic, cytoplasmic, as well as nuclear CA IX can be detected during IHC analysis of CA IX expression in tumor tissues and how the subcellular localization of CA IX may be related to the levels of the soluble form of CA IX.

Here, we detected membranous or membranous/cytoplasmic expression of CA IX in 66.67% of tCA IX+ cases and cytoplasmatic staining in 33.33% of tCA IX+ cases (Table 3). Cytoplasmic staining of CA IX occurred mainly in the luminal A subtype with grade II without association with necrosis, and in two cases of the HER2+ subtype with grade III, in which necrosis was present. It should be mentioned that in four out of eight of these cases we detected sCA IX plasma concentrations close to the cut off value and in two cases we did not detect sCA IX at all. Thus, cytoplasmic localization of tCA IX appears to be associated (albeit not significantly) with lower levels of sCA IX. 

In addition to IHC staining of CA IX bound to the cell membrane and/or cytoplasm, we also noted 18 cases (18.00%) of nuclear-localized CA IX. Similarly, nuclear CA IX was observed mostly in the luminal A and HER2+ molecular subtypes of BC, in cases with lower grading, positive necrosis, and ER- status (Appendix B, Table A1). In addition, we observed the highest levels of sCA IX in these patients (mean = 895.8 pg/mL) (Appendix B, Figure A2). Finally, as we documented in Appendix C, we detected the most CA IX-positive exosomes (detectable by our ELISA) in a patient with a very high plasma sCA IX level (1440.00 pg/mL) in whom nuclear CA IX staining was noted. This suggests, as we mentioned above, the connection of the tCA IX nuclear signal with the nucleo-cytoplasmic trafficking machinery that seems to be related to endocytosis, the product of which are also exosomes (i.e., higher levels of sCA IX). However, we need to verify these findings on a larger number of patients. 

When searching for an answer to the question why the amount of membrane localized CA IX in the tissue does not always correlate with sCA IX in the plasma, it is necessary to look at CA IX ectodomain shedding in the second (but no less important) line. Ectodomain cleavage is an important regulatory mechanism that controls the abundance and/or function of proteins bound to the cell membrane, as well as the molecular composition of the extracellular microenvironment, which, in turn, affect cell phenotype and intercellular signaling [46]. CA IX ectodomain (ECD) shedding is a regulated process that responds to hypoxia and acidosis, as well as cytotoxic compounds or chemotherapeutic drugs. The CA IX ECD can be cleaved by metalloproteinases ADAM17 [14] and, as proven recently, also ADAM10 [16]. Both genes are transcriptionally activated by hypoxia, but while ADAM10 seems to respond also to moderate hypoxia (0.5–5% O_2_), ADAM17 is transcriptionally induced by severe hypoxia (below 0.1% O_2_) [49,50]. The expression pattern of ADAM17 and ADAM10 is tissue-specific. For example, in invasive BC the expression of ADAM17 is significantly increased in high-grade tumors independent of tumor size, lymph node involvement, and ER status [51]. Whereas, higher levels of ADAM10 were found more frequently in high-grade and ER-negative early BC tumors, and are likely to be mainly involved in the progression of the basal subtype of BC (TNBC) [52]. However, the activity of metalloproteinases cleaving CA IX depends not only on the level of their expression in particular tissue, but also on the expression of their activators and especially inhibitors, which, for ADAM10, are the tissue inhibitors of metalloproteinases (TIMP) 1 and 3, while for ADAM17 they are TIMP 3 and 4 [53]. Therefore, it is possible that the reduced concentrations of sCA IX (below the cut off value) that we observed in TNBC cases with high expressions of membrane tCA IX (Table 3) may be related to strong upregulation of TIMP1 expression in TNBC patients, leading to the inhibition of CA IX shedding mediated by ADAM10 [53,54]. If this association were confirmed in the future, impaired shedding of CA IX would be one of the factors contributing to the more aggressive phenotype of TNBC. Because, like our colleagues have shown, shedding impairment does not alter CA IX function, but induces cancer-promoting changes in the extracellular proteome in terms of increased ability of tumor cells to migrate and form metastatic lesions [55]. 

The presence of sCA IX in the plasma of the control group and in patients without the presence of tCA IX is not as surprising as it might seem. Although CA IX expression is almost exclusively induced by the transcription factor HIF-1α during tumor hypoxia [5], hypoxia is not exclusively associated with cancer [56]. Hypoxia participates to a greater or lesser extent in the pathophysiology of many non-oncological diseases and is commonly noticed in multiple sclerosis, heart disease, and kidney, liver, lung, and inflammatory bowel diseases. An important category are also pathologies associated with pregnancy, preeclampsia, and the HELLP syndrome, in the development of which hypoxia participates [57]. Therefore, it is not surprising that CA IX, a reliable and stable protein controlled by HIF-1α, has been used as a marker for the early diagnosis of hypoxia-related diseases in some recent works. For example, elevated CA IX plasma levels have been identified in women with overt preeclampsia [58]. The possible role of CA IX in cardiovascular diseases associated with hypoxia was also recently pointed out by our group, when we detected the CA IX protein in the tissue (in 12 of 15 samples) and sCA IX in the plasma (in 13 of 15 samples) of patients with abdominal aortic aneurysm [59]. Recently, elevated levels of sCA IX have even been detected in the plasma of patients suffering from obstructive sleep apnea [60]. Cancer patients can and often do suffer from other diseases, including diseases associated with hypoxia. Therefore, we cannot exclude that the part of sCA IX in plasma may originate from other organs where hypoxic upregulation may occur due to another, non-oncological, hypoxia-related disease. However, in cancer patients, the only physiologically correct response to cancer-related hypoxia is the expression of only the full-length CA IX protein located at the cell membrane, representing the only CA IX variant with prognostic value. 

Considering all the above facts, it is extremely important to have validated diagnostic tools of high quality available for the reliable and specific detection of various tumor-associated forms of CA IX. Currently, there is a limited number of diagnostic IVD-certified kits available on the market for the detection of CA IX by IHC and sCA IX in serum/plasma. To the best of our knowledge, there are only two antibodies that are able to detect CA IX in formalin-fixed paraffin-embedded tissues (FFPE): a mouse MAb MRQ-54 (also known as M75), and a rabbit MAb EP161, both from Cell Marque^TM^ Tissue Diagnostics, Rocklin, California, United States. However, MRQ-54 is not available on the market outside the United States. The availability of certified IVD kits for the detection of soluble CA IX is also insufficient. We know of only one CA IX ELISA manufactured by Nuclea Diagnostics, Cambridge, MA, former Siemens Medical Solutions Diagnostics, Tarrytown, NY. These are also serious reasons why controlled and randomized clinical trials (except for those using G250-based isotope-labeled antibodies for PET/CT diagnosis [61,62]), which would confirm the clinical relevance of CA IX as prognostic and/or predictive biomarker, are not ongoing. 

Based on our results of IHC staining of CA IX, we can conclude that the diagnostic ability of MAb IV/18 is reliable, as it specifically detects all subcellular forms of CA IX protein in early breast cancer tissue. To demonstrate the importance of this antibody, we want to note that in addition to good detection properties, Mab IV/18, after binding to its epitope, is able to block the attachment of tumor cells to the extracellular matrix, and thus inhibit the spread and formation of metastases in vivo. Due to these abilities, MAb IV/18 was one of two mouse anti-CA IX antibodies selected for humanization with the intention to develop anti-CA IX targeted therapy [63]. Importantly, its humanized version, CA9hu-2, retained the properties of the parental antibody. 

We also show that our CA IX ELISA kit can be used for the sensitive and specific detection of the soluble form of CA IX, which consists of the shedded CA IX ectodomain, CA IX-positive exosomes, and probably also the alternatively spliced variant of CA IX protein. Although prognostic significance of sCA IX in metastatic breast cancer was clearly demonstrated [21], here, similarly to Schütze and colleagues [64], we could not show the clear association of sCA IX with prognosis of early breast cancers. Since TNBC patients with high tCA IX had lower sCA IX levels, low sCA IX levels seem to indicate a worse prognosis in TNBC. However, these findings need to be verified in a larger patient population, and as has already been shown by other authors [22,23], there is a high assumption that in early breast cancers, sCA IX will be more important in predicting the response to selected drugs, which, however, needs to be confirmed in randomized clinical trials.

We believe that this study, in which we presented and verified the functionality of two new diagnostic tools for the detection of tissue and soluble CA IX, will contribute to their introduction into scientific practice and their IVD certification, and ultimately, to the establishment of CA IX as a biomarker in clinical practice.

## 4. Materials and Methods

### 4.1. Study Design and Patient Population

One hundred and two patients were included in this single-institutional, cohort study between April 2019 and October 2022 at the Breast Unit of the 2nd Department of Gynecology and Obstetrics, University Hospital of Bratislava, Slovak Republic. The inclusion criteria were: patients with a core biopsy-proven primary operable early breast carcinoma without preoperative systemic therapy or radiotherapy. 

### 4.2. Ethics Statement

The study was approved by the Comenius University and University Hospital of Bratislava Ethics Committee, Statement No. EC/190/2019. Written informed consent was obtained from all patients.

The study involving healthy volunteers was approved by the Ethics Committee of Biomedical Research Center of the Slovak Academy of Sciences, Statement No. EK/BmV-03/2021. Written informed consent was obtained from all patients.

### 4.3. Surgical Procedures and Plasma Sample Collection

At the introduction to general anesthesia, immediately after the insertion of the cannula, 5 mL of peripheral venous blood was taken from the patient. The sample was immediately transported to the laboratory for further processing and plasma samples were then frozen at −80 °C. 

Lymphatic mapping and sentinel lymph nodes biopsy were the initial surgical procedures. The day before surgery, 2.0 mL of superparamagnetic iron oxide nanoparticles (Magtrace^®^, Endomagnetics Ltd., Cambridge, UK), were injected into the subareolar interstitial tissue. Before the axillary skin incision, magnetic count numbers from the skin were measured using a 2nd generation magnetic probe (Sentimag^®^, Endo-magnetics, Ltd., Cambridge, UK). All excided sentinel lymph nodes (SLNs) were sent to frozen section evaluation. In patients with 3 or more positive SLNs, an axillary dissection was performed. Breast saving therapy or radical mastectomy were applied according to established criteria [65]. In patients with impalpable BC, a two-view specimen radiography was performed to confirm the complete tumor excision and safe surgical margins.

### 4.4. Pathology

All surgical samples of breast tumors were marked with ink on their surface and fixed in 10% formalin solution for further evaluation through serial sectioning and histopathology. The SLNs were intraoperatively examined with hematoxylin/eosin staining and postoperatively with IHC. For IHC, monoclonal mouse anti-human cytokeratin antibody (clones AE1/AE3, code M3515, DAKO) was used. According to established histopathologic criteria [66], lymph node macro-metastases measured >2 mm; micro-metastases were defined as a focus of metastasis measuring >0.2 mm and ≤2 mm (pN1mi). Isolated tumor cells were defined as microscopic clusters and single cells measuring 0.2 mm or less (pN0i+). Immunoreactivity for HER2, ER, and PR receptors was studied by the VENTANA-BenchMark-XT computerized automated system, using the ultraView Universal DAB Detection Kit (ROCHE), which detects specific mouse and rabbit primary antibodies bound to an antigen in paraffin-embedded tissue sections. Intensity score for HER2 was defined in scale of 0 to 3 (0: none, 1: weak staining, 2: moderate staining, 3: strong staining). In cases with HER2 score 2+ (equivocal), an additional evaluation through silver DNA in situ hybridization was used for ultimate positive or negative results. The histological grade of tumors was evaluated by the Elston–Bloom and Richardson system [66]. According to histopathology results, the tumors were categorized to intrinsic subtypes and pathologic prognostic stage groups according to the *AJCC Cancer Staging Manual, 8th edition* [2]. The patient’s characteristics are summarized in Table 1.

### 4.5. Preparation of Monoclonal Antibody for Immunohistochemistry

For monoclonal antibody production, hybridoma cells (made in our laboratory earlier [25]) were cultured in Dulbecco’s Modified Eagle Medium (Biochrom GmbH, Berlin, Germany) and supplemented with 10% low IgG fetal bovine serum (FBS) (Biosera). Antibodies from the culture medium were purified using HiTrap MabSelect SuRe columns (GE Healthcare) by using the ÄKTA pure chromatography system (Cytiva, Marlborough, MA, USA). Purified antibodies were neutralized with 0,5M Tris-HCl buffer pH-9.0, and subsequently dialyzed into phosphate-buffered saline (PBS) pH-7.0 at 4 °C. Pool fractions were concentrated using an Amicon^®^ Ultra-15 Centrifugal Filter Unit (Merck Millipore, Burlington, MA, USA) and the concentration of antibodies was determined using a Thermo Scientific Pierce BCA Protein Assay Kit. 

Preparation of low IgG FBS: To remove bovine IgG from serum, inactivated FBS was filtered through a 0.45 µm filter (Merck Millipore, USA), applied to a HiTrap MabSelect SuRe column (GE Healthcare), and purified using the ÄKTA pure chromatography system (Cytiva, USA). IgG antibodies remained trapped on the column. Flow-through FBS was filtered through a 0.22 µm filter (Merck Millipore, USA) and frozen at −20 °C until use.

### 4.6. CA IX Immunohistochemistry 

CA IX expression was detected by IHC using the purified MAb IV/18 (1mg/mL). Briefly, deparaffinized slides were rehydrated. Endogenous peroxidase activity and non-specific staining were blocked using EnVision™ FLEX Peroxidase-Blocking Reagent (DAKO). Slides were incubated for 60 min at room temperature with the aforementioned primary antibody diluted to 1:100 in REAL Antibody Diluent (DAKO) and immunostained using an anti-mouse immunoperoxidase polymer (EnVision FLEX/HRP; DAKO) for 30 min at room temperature. The reaction was visualized with a 3,3’-diaminobenzidine substrate-chromogen solution (Envision™ FLEX Substrate Working Solution, DAKO) for 5 min, and slides were counter-stained with hematoxylin (EnVision™ FLEX Hematoxylin, DAKO). Antibodies M75 (dilution 1/1000) and rabbit monoclonal antibody EP161 (dilution 1/100; Cell Marque^TM^ Tissue Diagnostics, Rocklin, California, United States) were used as controls in the same procedure manner. Clear cell renal cell carcinoma tissue was used as a positive control. As a negative control, the same tumor tissue was used, but omitting the primary antibody from the staining protocol.

The following characteristics were assessed and recorded for each patient case:

- Intensity of CA IX staining scored as (+), (++), and (+++);
- Relative number of CA IX positively stained cells: less than 10%, 11–50%, and more than 50% per field of view;
- Subcellular localization of CA IX antigen: membrane, mixed membrane/cytoplasmic, cytoplasmic, or nuclear.

The resulting CA IX score for the qualitative and quantitative assessment of tissue CA IX was determined as follows:

1. Low—membrane or mixed membrane/cytoplasmic or cytoplasmic subcellular localization, percentage of positive cells less than 10%, and at the same time, intensity of staining (+);
2. Focal—membrane or mixed membrane/cytoplasmic or cytoplasmic subcellular localization, percentage of positive cells 11–50%, and at the same time, intensity of staining (+/++) or (++);
3. High—membrane or mixed membrane/cytoplasmic subcellular localization, percentage of positive cells more than 50%, and intensity of staining (+++);
4. Negat—nuclear subcellular localization regardless of the percentage of positive cells and staining intensity, and negative CA IX staining.

The evaluation of CA IX expression in BC tissues was performed independently by two pathologists.

### 4.7. CA IX ELISA

For the quantitative determination of sCA IX concentration in plasma, the ELISA kit originally developed by BMC SAS was used and modified to its current form in cooperation with MABPRO, a.s. The assay was performed at room temperature. The microplate wells were coated overnight with 100 μL/well of an anti-CA IX ectodomain (ECD), MAb VII/32 [25]. The capture antibody was diluted in PBS to a concentration of 8 μg/mL. Non-specific binding was blocked with MDB solution (BioLab Assays s.r.o., Brno, Czech Republic) for 1 h, and washed three times with PBS + 0.05% Tween-20 (PBST). Serum samples diluted three times in MDB solution were added in a volume of 100 μL/well and incubated for 1 h. After washing three times with PBST, the peroxidase-conjugated detection MAb IV/18 (0.5 μg/mL in StabilZyme™ HRP Conjugate Stabilizer (Surmodics IVD, Inc., Eden Prairie, MN, USA)) was added in a volume of 100 μL/well and incubated for 1 h. Finally, the microplate was washed three times with PBST and the substrate solution SeramunBlau^®^ fast2 (Seramun Diagnostica GmbH, Germany) was added in volume of 100 μL/well for 20 min in the dark. The reaction was stopped by adding 50 μL of 0.2M H_2_SO_4_ (Biovendor-LM, Brno, Czech Republic) to each well. Absorbance was measured at 450 nm. Concentrations of sCA IX were quantitated based on a calibration curve obtained using the CA IX ECD protein (amino acids 38–406) [25], which was used as a standard. Each sample, standard, and control was analyzed in duplicate. Plasma samples from 40 apparently healthy individuals (mean age 60 years; range 57–71 years) were used as a control in the ELISA assay.

### 4.8. In Silico Analysis

The Tumor online Prognostic analysis Platform (ToPP) [30], which collects multi-omics and clinical data from 55 types of tumor datasets from The Cancer Genome Atlas (TCGA), International Cancer Genome Consortium (ICGC), and the Clinical Proteomic Tumor Analysis Consortium (CPTAC), was used for *CA9* gene expression analysis. The tumor versus normal expression, stage distribution, average age, and overall survival were analyzed using the TCGA-BRCA dataset. Additionally, Breast Cancer Gene-Expression Miner (bc-GenExMiner; [31]) v4.9, a statistical mining tool of published annotated breast cancer transcriptomic data (DNA microarrays and RNA-seq), was used to explore *CA9* gene expression in breast cancer. Results of *CA9* expression, according to hormone receptor status (ER, PR) and HER2, as well as prognostic grades and index, were analyzed and are presented in either box and whiskers or violin graphs.

### 4.9. Statistical Analyses

Statistical analyses were conducted using the GraphPad Prism (v9) software and/or MedCalc^®^ Statistical Software version 20.2 (MedCalc Software Ltd., Ostend, Belgium; https://www.medcalc.org (accessed on 1 October 2022); 2022). Analysis of *CA9* gene expression in the BC was performed using the Tumor online Prognostic analysis Platform (ToPP) and the Breast Cancer Gene-Expression Miner v4.9. A *p*-value of *<0.05* was considered statistically significant. 

To evaluate the association between clinicopathologic categorical variables and CA IX expression in tumor tissue, chi-squared and/or Fisher’s exact tests were performed (Table 2 and Table A1 in Appendix B). To evaluate the association between sCA IX below and above the cut off value with the low, focal, and high tCA IX groups, a chi-squared test was performed (Figure 4). 

To determine the optimal cut off value of both CA IX ELISA and R&D ELISA, ROC curve analysis was performed using MedCalc^®^ Statistical Software version 20.2 (Figure 2A and Figure A7 in Appendix E). The optimal cut off value was identified based on Youden’s criterion maximizing Youden’s J statistic (sensitivity + specificity). 

A Mann–Whitney test for independent samples was used to evaluate the differences in sCA IX levels between the groups of healthy and tested BC patients (Figure 2B,C and Figure A7C in Appendix E).

An unpaired *t*-test was used to evaluate the differences in sCA IX levels in the plasma of patients with CA IX-positive versus CA IX-negative tissue (Figure 3 and Figure A10 in Appendix E). 

A Kruskal–Wallis test with Dunn’s post hoc test was used to evaluate the statistical significance of differences in sCA IX levels of patients divided based on CA IX score into: low, focal, and high tCA IX groups (Figure 4 and Figure A11 in Appendix E).

A Kruskal–Wallis test with Dunn’s post hoc test was used to evaluate the differences in sCA IX levels between patients who are CA IX-positive, CA IX-negative, and CA IX-negative with nuclear CA IX signal in tumor tissue (Figure A2 in Appendix B).

The Wilcox test was used to evaluate the differences between the two groups (Figure 5A–C and Figure 6A–C). To evaluate the association between the *CA9* expression and survival in BC, the univariate analysis presented as a Kaplan–Meier survival plot was performed (Figure 5D). Significant differences between groups were assessed by Welch’s test (Figure 6D,E and Figure A6 in Appendix D), and a Dunnett–Tukey–Kramer’s test was used for each pairwise comparison (Figure 6D,E and Figure A6 in Appendix D). 

A dot and line diagram of pairwise comparison (Figure A8 in Appendix E) and scatter diagram (Figure A9 in Appendix E) of individual patients’ sCA IX concentrations measured by both CA IX ELISA and R&D ELISA were constructed by using the MedCalc Software. The degree of relationship between sCA IX concentrations of individual patients measured by CA IX ELISA versus R&D ELISA was determined using rank correlation.

## Figures and Tables

**Table 1:** Patient characteristics.

No. of Evaluated Patients	100	
**Age**–median (range)	**57.5** (34–85)	
** Menopausal status **		
Pre-menopause	26	26%
Post-menopause	73	73%
Not applicable—male	1	1%
** Grade **		
I	6	6%
II	57	57%
III	36	36%
DCIS	1	1%
** T stage **		
pTis	3	3%
pT1	56	56%
pT2	37	37%
pT3	3	3%
pT4	1	1%
** N status **		
pN0	64	64%
pN1	21	21%
pN2	12	12%
pN3	3	3%
** ER **		
Positive	86	86%
Negative	14	14%
** PR **		
Positive	81	81%
Negative	19	19%
** HER2 **		
Positive	16	16%
Negative	84	84%
** Tumor necrosis **		
Negative	78	78%
Positive (+)	14	14%
Positive (+++)	1	1%
Micro	6	6%
Comedo necrosis	1	1%

**Table 2:** Association of tumor tissue CA IX expression with clinicopathological characteristics.

Variables (n)	CA IX+ (n = 24)	CA IX- (n = 76)	*p*-Value	CA IX Score			*p*-Value
				Low	Focal	High	
** Subtype **							
Luminal A (54)	9	45	* 0.0115 *	3	4	2	* ns *
Luminal B-like (18)	5	13		0	2	3	
HER2-enriched (16)	3	13		1	1	1	
TNBC (9)	6	3		2	1	3	
DCIS (3)	1	2		1	0	0	
** Grade **							
I/II (63)	10	53	* 0.0146 *	4	5	1	* 0.0060 *
III (36)	14	22		3	3	8	
** pT **							
pT1 (56)	9	47	* ns *	2	4	3	* ns *
pT2-4 (41)	14	27		4	4	6	
** pN **							
pN0 (64)	14	50	* ns *	2	6	6	* ns *
pN1-3 (36)	10	26		5	2	3	
** ER **							
Positive (86)	17	69	* 0.0019 *	4	7	6	* ns *
Negative (14)	7	7		3	1	3	
** PR **							
Positive (81)	17	64	* ns *	4	7	6	* ns *
Negative (19)	7	12		3	1	3	
** HER2 **							
Positive (16)	3	13	* ns *	1	1	1	* ns *
Negative (84)	21	63		6	7	8	
** Tumor necrosis **							
Positive (21)	11	10	* 0.0015 *	4	3	4	* 0.0062 *
Negative (78)	13	65		3	5	5	

For the evaluation of the relation between CA IX+ expression and clinicopathological characteristics, namely grade, pT, pN, ER, PR, HER2, and tumor necrosis, Fisher’s exact test was used. For the evaluation of the relation between CA IX+ expression and molecular subtypes of BC, a chi-squared test was used. For the evaluation of the relation between CA IX score and clinicopathological characteristics, a chi-squared test was used. A *p*-value of <0.05 was considered statistically significant. n—number of patients; *ns*—non-significant.

**Table 3:** Summary table of clinicopathological characteristics of patients with tissue-positive CA IX expression and soluble CA IX levels in their plasma.

CA IX Score	Clinicopathological Variables							tCA IX			sCA IX
	pT	pN	Grade	ER/PR	HER2	Molecular Subtype	Necrosis	L	I	% Cells	pg/mL
**Low**	2c	1a	III	-/-	-	TNBC	+	M + C	+	<10%	954.0
	1c	2a	III	-/-	-	TNBC	0	M + C	+	<10%	1392.0
	4b (m)	3a	II	+/+	-	Luminal A	+	M + C	+	<10%	1662.0
	1b	0(sn)	I	+/+	-	Luminal A	0	M	+	<10%	2592.0
	2 (m)	1a	II	+/+	-	Luminal A	0	C	+ / ++	<10%	750.0
	is (m)	0(sn)	II	+/+	-	DCIS	+	C	+	<10%	978.0
	*2*	*1a*	*III*	*-/-*	*3+*	*HER2+*	*+*	*C*	*+*	*<10%*	*552.0*
**Focal**	3	0	III	-/-	-	TNBC	+++	M+C	++	11–50%	813.0
	2	2a	II	+/+	-	Luminal B-like	+	M + C	++	11–50%	951.0
	1c	0(sn)	II	+/+	-	Luminal A	0	C	++	11–50%	726.0
	1c	0(sn)	II	+/+	-	Luminal A	0	C	+ / ++	11–50%	579.0
	2 (m)	0	III	+/+	+	HER2+	+	C	+ / ++	11–50%	1041.0
	*2*	*0(sn)*	*II*	*+/+*	*-*	*Luminal A*	*0*	*M*	*++*	*11–50%*	*487.5*
	*1a*	*0(sn)*	*II*	*+/+*	*-*	*Luminal A*	*0*	*C*	*++*	*11–50%*	*0.0*
	*1b*	*2a*	*III*	*+/+*	*-*	*Luminal B-like*	*0*	*C*	*++*	*11–50%*	*0.0*
**High**	2	0	III	+/+	-	Luminal B-like	0	M	+++	>50%	588.0
	2	1a	III	+/+	-	Luminal B-like	0	M	+++	>50%	906.0
	1c	0 (sn)	III	+/+	-	Luminal B-like	+	M	+++	>50%	903.0
	2	2a	III	+/+	+	HER2+	+	M	+++	>50%	615.0
	1c	0 (sn)	III	+/+	-	Luminal A	0	M	+++	>50%	1431.0
	1c	1a	II	+/+	-	Luminal A	0	M	+++	>50%	792.0
	*2*	*0*	*III*	*-/-*	*-*	*TNBC*	*+*	*M*	*+++*	*>* *50%*	*564.0*
	*2*	*0(sn)*	*III*	*-/-*	*-*	*TNBC*	*0*	*M*	*+++*	*>* *50%*	*459.0*
	*2 (m)*	*0*	*III*	*-/-*	*-*	*TNBC*	*+*	*M*	*+++*	*>* *50%*	*210.6*

The table shows the clinicopathological characteristics of patients with tCA IX+, divided according to the CA IX score into low, focal, and high groups, and the concentrations of sCA IX that were detected in their plasma. L—subcellular localization of tissue CA IX; I—intensity of CA IX staining; % cells—the number of CA IX-positive cells expressed in %. Patients with sCA IX levels below the cut off value are highlighted in italics.

**Figure A3:**
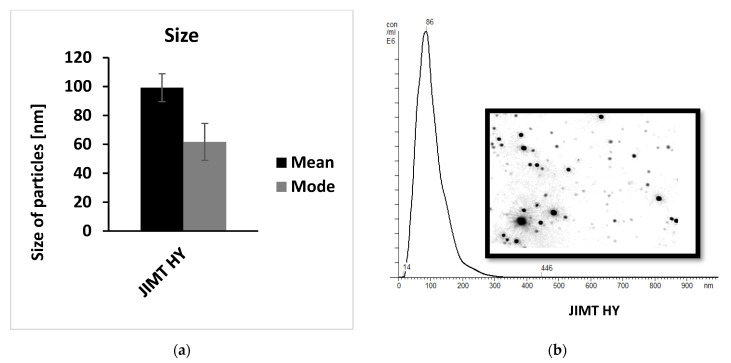
Characterization of exosomes isolated from hypoxic JIMT-1 cells. (**a**) The size distribution was analyzed by NTA using NanoSight NS500 (for each sample 5x 60-second run); (**b**) representative nanoparticle tracking analysis (NTA) of isolated exosomes diluted with DPBS.

**Figure A4:**
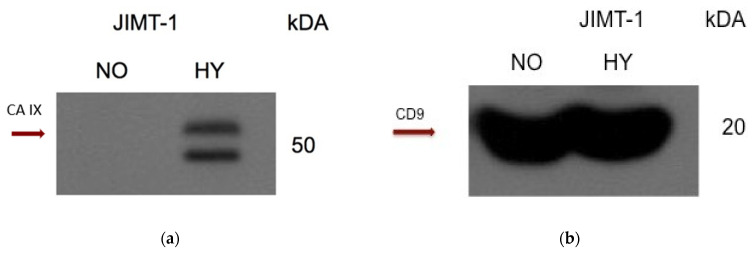
Characterization of exosomes from JIMT cells grown in hypoxia for 48 h. Expression of CA IX (**a**) and CD9 (specific marker of exosomes) (**b**) proteins was analyzed by Western blotting using specific antibodies.

**Figure A5:**
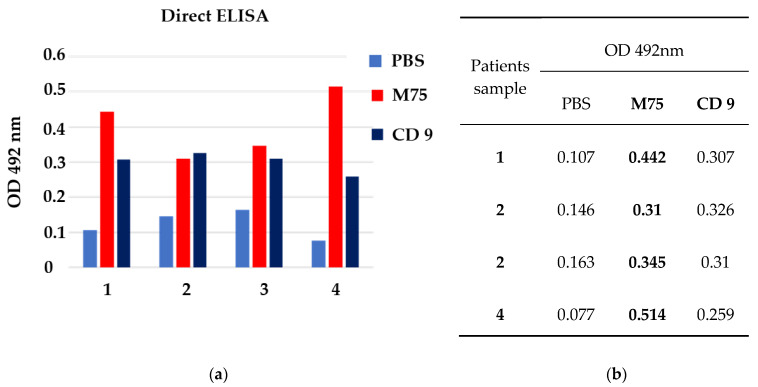
Characterization of exosomes isolated from the plasma of four patients. (**a**) Expression of CA IX and CD9 (specific marker of exosomes) analyzed by direct ELISA; (**b**) OD values at 492 nm.

## Data Availability

The datasets used and analyzed during the current study are available from both corresponding authors on reasonable request.

## Abbreviations

ASCO	American Society of Clinical Oncology
AUC	An area under the curve
BC	Breast cancer
BCI	Breast Cancer Index
BRCA	Breast invasive carcinoma
BRCA 1/2	Breast cancer gene 1/2
CA IX	Carbonic anhydrase IX
*CA9*	Carbonic anhydrase 9 gene or transcript
ccRCC	Clear cell renal cell carcinoma
CI	Confidence interval
DCIS	Ductal carcinoma in situ
DSF	Disease-free survival
DSS	Disease-specific survival
ECD	Ectodomain of CA IX
ELISA	An enzyme-linked immuno-sorbent assay
ER	Estrogen receptor
FBS	Fetal bovine serum
FFPE	Formalin-fixed paraffin-embedded tissues
HELLP	Hemolysis, elevated liver enzymes and low platelets syndrome
HER2	Human epidermal growth factor 2 receptor
HR	Hazard ratio
IHC	Immunohistochemistry
IVD	In vitro diagnostics
KM	Kaplan–Meier
MAb	Monoclonal antibody
MSF	Metastasis-free survival
NPI	Nottingham prognostic index
*ns*	Non-significant
NTA	Nanoparticle tracking analysis
OS	Overall survival
PBS	Phosphate-buffered saline
PFS	Progression-free survival
PG	Proteoglycan
PR	Progesterone receptor
ROC	A receiver operating characteristic curve
SBR	Scarff–Bloom–Richardson
sCA IX	Soluble form of CA IX
SLNs	Sentinel lymph nodes
tCA IX	Tissue CA IX
TCGA	The Cancer Genome Atlas
TNBC	Triple-negative breast cancer
TNM	Tumor, node and metastasis staging
ToPP	Tumor online Prognostic analysis Platform

## Appendix B

**Nuclear CA IX in tumor tissue and corresponding sCA IX in plasma of BC patients.**

**Table A1:** Association of nuclear CA IX expression with clinicopathological characteristics.

Variables (n)	CA IX+ (n = 24)	CA IX- (n = 76)		*p*-Value
		WS (n = 58)	NS (n = 18)	
**Subtype**				
Luminal A (54)	9	32	13	*0.0142*
Luminal B-like (18)	5	13	0	
HER2-enriched (16)	3	9	4	
Triple-negative (9)	6	2	1	
DCIS (3)	1	2	0	
**Grade**				
I/II (63)	10	40	13	*0.0363*
III (36)	14	17	5	
**pT**				
pT1 (56)	9	34	13	*ns*
pT2-4 (41)	14	22	5	
**pN**				
pN0 (64)	14	39	11	*ns*
pN1-3 (36)	10	19	7	
**ER**				
Positive (86)	17	54	15	*0.0023*
Negative (14)	7	4	3	
**PR**				
Positive (81)	17	49	15	*ns*
Negative (19)	7	9	3	
**HER2**				
Positive (16)	3	9	4	*ns*
Negative (84)	21	49	14	
**Tumor necrosis**				
Positive (21)	11	6	4	*0.0018*
Negative (78)	13	51	14	

For the evaluation of the relation between CA IX nuclear expression and clinicopathological characteristics, a chi-squared test was used. (n)—number of patients; WS—IHC staining without CA IX signal; NS—IHC staining with CA IX nuclear signal; *ns*—non-significant.

## Appendix C

**Exosomes released from breast cancer cells contain CA IX and can be detected in patient plasma with CA IX ELISA.**

### Appendix C.1. Materials and Methods

#### Appendix C.1.1. Isolation of Exosomes

Exosomes were isolated from 5 × 10^7^ of JIMT-1 cells (ACC 589, DSMZ-German Collection of Microorganisms and Cell Cultures GmbH, Germany), cultivated in DMEM supplemented with 5% fetal bovine serum, exosome depleted (Thermo Fisher Scientific Inc.) for the first 24 h, followed by cultivation under hypoxic conditions 1% O_2_ for 48 h in a hypoxia workstation (Ruskin Technologies). Then, the medium was centrifuged at 300× *g* for 5 min, and at 1200× *g* for 10 min to remove cell debris, and filtered through a 0.22 µm filter (Merck Millipore, USA). The pre-cleared medium was concentrated using 100 kDa MWCO Amicon Ultra Centrifugal Filter (Merck Millipore, USA) at 1200× *g*. Finally, exosomes were isolated using a Total Exosome Isolation Kit (Invitrogen/Thermo Fisher Scientific, USA) during an overnight incubation at 4 °C, sedimented by centrifugation at 10,000× *g* for 1 h, and diluted in DPBS (Thermo Fisher Scientific Inc.).

Plasma exosomes were isolated using an Invitrogen™ Total Exosome Isolation Kit (from plasma) according to the manufacturer’s instructions.

#### Appendix C.1.2. Nanoparticle Tracking Analysis

For the rapid in vitro measurements of exosomes, we performed nanoparticle tracking analysis (NTA) using NanoSight NS500 equipped with an sCMOS Trigger camera and a 405 nm laser (Malvern Instruments Ltd., Malvern, UK). NTA utilizes the properties of both light scattering and Brownian motion to obtain the size distribution and concentration measurements of particles in liquid suspension. The measured data were analyzed using the NTA2.3 analytical software. Each sample was diluted in PBS before the measurements to optimize the number of particles. Samples were measured in quintuplicate in 60-second videos with manual shutter and gain adjustments.

#### Appendix C.1.3. CA IX and CD9 direct ELISA

Microplate wells (flat) were coated overnight at 37 °C with 50 μL of exosomes as an antigen (diluted in DPBS) and allowed to dry. After blocking with 10% FCS in PBS + 0.05% Tween-20 for 2 h at RT, the coated wells were incubated with 50 μL of anti-CA IX mouse MAb M75 (hybridoma medium) or anti-CD9 rabbit antibody (CD9 (H-110) sc-9148, Santa Cruz Biotechnology, 1:100) for 2 h at RT. After washing 3x with PBS + 0.05% Tween-20, 50 μL of peroxidase-labeled pig anti-mouse IgG (Sigma-Aldrich, diluted 1:5000 in PBS with 10% FCS for 2 h) or peroxidase-labeled anti-rabbit IgG (Sigma-Aldrich, diluted 1:5000 in PBS with 10% FCS for 2 h) was used as detector. After washing 3x with PBS + 0.05% Tween-20 the reaction was visualized by adding 50 μL of substrate prepared as follows: 10μg ortho-phenylene-diamine (Sigma, P9020) and 10 μL H_2_O_2_ in 10 mL citrate buffer pH 5.0. After 5–7 min incubation in the dark, 50 μL of stop solution (2M H_2_SO_4_) were added. Reaction was measured at 492 nm.

#### Appendix C.1.4. Western blot

Isolated exosomes were separated by SDS–polyacrylamide gel (10%) electrophoresis. Afterwards, the proteins were transferred onto a PVDF membrane and probed with the following antibodies: anti-CA IX MAb M75 (hybridoma medium 1:3 in 5% non-fat dry milk with 0.2% Nonidet P40 in PBS, 1 h, RT) and anti-CD9 (cell signaling, D801A, rabbit Mab 1:1000 in 3% BSA in TBS-T buffer, 1 h, RT), and visualized by anti-mouse-HRP or anti-rabbit-HRP (Sigma-Aldrich, 1:5000 in 5% non-fat dry milk with 0.2% Nonidet P40/0.1% Tween20 in PBS, 1 h, RT). Protein bands were detected using an enhanced chemiluminescence kit (GE Healthcare Bio-Sciences).

### Appendix C.2. Results

For the isolation of exosomes, we employed cell lines JIMT-1 naturally expressing CA IX under hypoxic conditions. Exosomes isolated under hypoxic treatment were characterized by NTA. NTA was carried out using NanoSight NS500 confirming the size distribution with a mean = 99 nm and an average mode = 62 for hypoxic exosomes (Figure A3).

The expression of CA IX in exosomes isolated from JIMT-1 cells was also determined using Western blotting. Figure A4a shows the level of CA IX in exosomes detected in hypoxia. The correct isolation of exosomes was verified through the presence of a specific marker of exosomes—CD9 (Figure A4b).

To confirm that our ELISA, which was used to determine sCA IX in the plasma of BC patients, is able to detect CA IX-positive exosomes, we used it to evaluate the concentration of the CA IX-positive/negative exosomes isolated from JIMT-1 cells. The concentration of CA IX in the fraction of CA IX-positive exosomes was more than 4000 pg/mL.

Finally, to prove that the plasma of patients included in our study contains CA IX-positive exosomes, we isolated exosomes from the plasma of four patients with determined sCA IX positivity. The presence of CA IX and the exosome marker CD9 in exosomes isolated from the patients’ plasma was analyzed using direct ELISA (Figure A5). We proved the presence of CA IX-positive exosomes in patient samples. Moreover, we found that patients (four) with high sCA IX levels in their plasma (1440.00 pg/mL) and CA IX-negative tissue with nuclear CA IX signal had the greatest number of CA IX-positive exosomes.

## Appendix E

**Detection of sCA IX in plasma using Quantikine^®^ Human Carbonic Anhydrase IX Immunoassay**

The sCA IX levels in the patient’s plasma were also detected using a commercial kit intended for research use only (RUO)—Quantikine^®^ Human Carbonic Anhydrase IX Immunoassay (R&D Systems; DCA900) (R&D ELISA). We performed the ELISA test according to the manufacturer’s instructions. We followed the same procedure for evaluating the results of the R&D ELISA as we did for evaluating the results of our CA IX ELISA. First, we compared the sCA IX plasma level of BC patients (n = 65) with that of apparently healthy volunteers (n = 40) and identified the optimal cut off based on Youden’s criterion maximizing Youden’s J statistic (sensitivity + specificity). As shown by a receiver operating characteristic (ROC) curve in Figure A7 (AUC= 0.872: 95% CI: [0.5115 to 0.7365]) using this test with cut off of 37.8 pg/mL, according to the Youden’s statistic, can yield sensitivity of 75.4% and specificity of 90%. The difference between the compared groups (Control versus BC patients) was statistically significant *p < 0.001*.

We performed a pairwise comparison and correlation analysis of sCA IX concentrations measured by our CA IX ELISA kit and the R&D ELISA kit. As shown in the pairwise comparison dot and line graph (Figure A8) and in the scatter plot of correlation degree (Figure A9), the sCA IX concentrations measured by both kits are correlated.

The scatter diagram of sCA IX concentrations of individual patients measured by both ELISA tests shows a strong correlation. The degree of relationship between R&D ELISA and CA IX ELISA measurements was determined using rank correlation with the following result: Spearman’s coefficient of rank correlation = *0.800*, Kendall’s Tau = *0.640*, significance level *p < 0.0001*.

Similar to our ELISA, when we examined sCA IX in plasma using the R&D ELISA, we found that tCA IX+ patients did not have significantly higher sCA IX levels compared to tCA IX- patients (Figure A10). The average concentration of sCA IX in patients with tCA IX+ was 86.8 pg/mL, while in patients with tCA IX- it was 75.52 pg/mL. Of the 21 patients with tCA IX+, 16 had sCA IX higher than the cut off value. The remaining five had sCA IX below the cut off value, and in one of them we did not detect the presence of sCA IX at all. Out of 44 patients with tCA IX-, up to 33 patients had sCA IX above the cut off value. Differences between sCA IX levels in tCA IX+ and tCA IX- groups were not statistically significant (*p = 0.5613).*

Analysis of sCA IX levels in patients divided by CA IX score into low, focal, and high tCA IX groups confirmed what we detected via our ELISA kit, namely that patients with low tCA IX had the highest plasma sCA IX levels (Figure A11). Most patients from both groups, high tCA IX (five of eight) and focal tCA IX (five of seven), also had sCA IX levels above the cut off. Statistical analysis did not confirm significant differences between groups (*p = 0.7221*).

## References

[1] Arnold M., Morgan E., Rumgay H., Mafra A., Singh D., Laversanne M., Vignat J., Gralow J.R., Cardoso F., Siesling S. (2022). Current and future burden of breast cancer: Global statistics for 2020 and 2040. Breast.

[2] Amin M.B., Edge S.B., Greene F.L., Byrd D.R., Brookland R.K., Washington M.K., Gershenwald J.E., Compton C.C., Hess K.R., Sullivan D.C. (2017). AJCC Cancer Staging Mmanual.

[3] Varga Z., Sinn P., Seidman A.D. (2019). Summary of head-to-head comparisons of patient risk classifications by the 21-gene Recurrence Score^®^ (RS) assay and other genomic assays for early breast cancer. Int. J. Cancer.

[4] Andre F., Ismaila N., Allison K.H., Barlow W.E., Collyar D.E., Damodaran S., Henry N.L., Jhaveri K., Kalinsky K., Kuderer N.M. (2022). Biomarkers for Adjuvant Endocrine and Chemotherapy in Early-Stage Breast Cancer: ASCO Guideline Update. J. Clin. Oncol..

[5] Wykoff C.C., Beasley N.J.P., Watson P.H., Turner K.J., Pastorek J., Sibtain A., Wilson G.D., Turley H., Talks K.L., Maxwell P.H. (2000). Hypoxia-inducible Expression of Tumor-associated Carbonic Anhydrases. Cancer Res..

[6] Pastorek J., Pastorekova S. (2015). Hypoxia-induced carbonic anhydrase IX as a target for cancer therapy: From biology to clinical use. Semin. Cancer Biol..

[7] Pastorekova S., Gillies R.J. (2019). The role of carbonic anhydrase IX in cancer development: Links to hypoxia, acidosis, and beyond. Cancer Metastasis Rev..

[8] van Kuijk S.J.A., Yaromina A., Houben R., Niemans R., Lambin P., Dubois L.J. (2016). Prognostic Significance of Carbonic Anhydrase IX Expression in Cancer Patients: A Meta-Analysis. Front. Oncol..

[9] Brennan D.J., Jirstrom K., Kronblad A., Millikan R.C., Landberg G., Duffy M.J., Rydén L., Gallagher W.M., O’Brien S.L. (2006). CA IX is an independent prognostic marker in premenopausal breast cancer patients with one to three positive lymph nodes and a putative marker of radiation resistance. Clin. Cancer Res..

[10] Sowa T., Menju T., Chen-Yoshikawa T.F., Takahashi K., Nishikawa S., Nakanishi T., Shikuma K., Motoyama H., Hijiya K., Aoyama A. (2017). Hypoxia-inducible factor 1 promotes chemoresistance of lung cancer by inducing carbonic anhydrase IX expression. Cancer Med..

[11] Moreno-Acosta P., Vallard A., Carrillo S., Gamboa O., Romero-Rojas A., Molano M., Acosta J., Mayorga D., Rancoule C., Garcia M.A. (2017). Biomarkers of resistance to radiation therapy: A prospective study in cervical carcinoma. Radiat. Oncol..

[12] Ward C., Meehan J., Gray M., Kunkler I., Langdon S., Argyle D. (2018). Carbonic Anhydrase IX (CAIX), Cancer, and Radiation Responsiveness. Metabolites.

[13] Ilardi G., Zambrano N., Merolla F., Siano M., Varricchio S., Vecchione M., Rosa G., Mascolo M., Staibano S. (2014). Histopathological Determinants of Tumor Resistance: A Special Look to the Immunohistochemical Expression of Carbonic Anhydrase IX in Human Cancers. Curr. Med. Chem..

[14] Zatovicova M., Sedlakova O., Svastova E., Ohradanova A., Ciampor F., Arribas J., Pastorek J., Pastorekova S. (2005). Ectodomain shedding of the hypoxia-induced carbonic anhydrase IX is a metalloprotease-dependent process regulated by TACE/ADAM17. Br. J. Cancer.

[15] Vidlickova I., Dequiedt F., Jelenska L., Sedlakova O., Pastorek M., Stuchlik S., Pastorek J., Zatovicova M., Pastorekova S. (2016). Apoptosis-induced ectodomain shedding of hypoxia-regulated carbonic anhydrase IX from tumor cells: A double-edged response to chemotherapy. BMC Cancer.

[16] Zatovicova M., Kajanova I., Takacova M., Jelenska L., Sedlakova O., Labudova M., Pastorekova S. (2022). ADAM10 mediates shedding of carbonic anhydrase IX ectodomain non-redundantly to ADAM17. Oncol. Rep..

[17] Horie K., Kawakami K., Fujita Y., Sugaya M., Kameyama K., Mizutani K., Deguchi T., Ito M. (2017). Exosomes expressing carbonic anhydrase 9 promote angiogenesis. Biochem. Biophys. Res. Commun..

[18] Logozzi M., Capasso C., Di Raimo R., Del Prete S., Mizzoni D., Falchi M., Supuran C.T., Fais S. (2019). Prostate cancer cells and exosomes in acidic condition show increased carbonic anhydrase IX expression and activity. J. Enzym. Inhib. Med. Chem..

[19] Wind T.C., Messenger M.P., Thompson D., Selby P.J., Banks R.E. (2011). Measuring carbonic anhydrase IX as a hypoxia biomarker: Differences in concentrations in serum and plasma using a commercial enzyme-linked immunosorbent assay due to influences of metal ions. Ann. Clin. Biochem. Int. J. Lab. Med..

[20] Ho D., Huang J., Chapman J.-A.W., Leitzel K., Ali S.M., Shepherd L., Parulekar W.R., Ellis C.E., Crescnzo R.J., Zhu L. (2017). Impact of serum HER2, TIMP-1, and CAIX on outcome for HER2+ metastatic breast cancer patients: CCTG MA.31 (lapatinib vs. trastuzumab). Breast Cancer Res. Treat..

[21] Müller V., Riethdorf S., Rack B., Janni W., Fasching P.A., Solomayer E., Aktas B., Kasimir-Bauer S., Zeitz J., Pantel K. (2011). Prospective evaluation of serum tissue inhibitor of metalloproteinase 1 and carbonic anhydrase IX in correlation to circulating tumor cells in patients with metastatic breast cancer. Breast Cancer Res. BCR.

[22] Brown-Glaberman U., Marron M., Chalasani P., Livingston R., Iannone M., Specht J., Stopeck A.T. (2016). Circulating Carbonic Anhydrase IX and Antiangiogenic Therapy in Breast Cancer. Dis. Markers.

[23] Janning M., Müller V., Vettorazzi E., Cubas-Cordova M., Gensch V., Ben-Batalla I., Zu Eulenburg C., Schem C., Fasching P.A., Schnappauf B. (2019). Evaluation of soluble carbonic anhydrase IX as predictive marker for efficacy of bevacizumab: A biomarker analysis from the geparquinto phase III neoadjuvant breast cancer trial. Int. J. Cancer.

[24] Takacova M., Kajanova I., Kolarcikova M., Lapinova J., Zatovicova M., Pastorekova S. (2021). Understanding metabolic alterations and heterogeneity in cancer progression through validated immunodetection of key molecular components: A case of carbonic anhydrase IX. Cancer Metastasis Rev..

[25] Zatovicova M., Tarabkova K., Svastova E., Gibadulinova A., Mucha V., Jakubicková L., Biesová Z., Rafajová M., Ortova Gut M., Parkkila S. (2003). Monoclonal antibodies generated in carbonic anhydrase IX-deficient mice recognize different domains of tumour-associated hypoxia-induced carbonic anhydrase IX. J. Immunol. Methods.

[26] Pastoreková S., Závadová Z., Košťál M., Babušíková O., Závada J. (1992). A novel quasi-viral agent, MaTu, is a two-component system. Virology.

[27] Bartošová M., Parkkila S., Pohlodek K., Karttunen T.J., Galbavý Š., Mucha V., Harris A.L., Pastorek J., Pastoreková S. (2002). Expression of carbonic anhydrase IX in breast is associated with malignant tissues and is related to overexpression of c-erbB2: Carbonic anhydrase IX and c-erbB2 in breast cancer. J. Pathol..

[28] Chia S.K., Wykoff C.C., Watson P.H., Han C., Leek R.D., Pastorek J., Gatter K.C., Ratcliffe P., Harris A.L. (2001). Prognostic significance of a novel hypoxia-regulated marker, carbonic anhydrase IX, in invasive breast carcinoma. J. Clin. Oncol..

[29] Kalavska K., Chovanec M., Zatovicova M., Takacova M., Gronesova P., Svetlovska D., Baratova M., Miskovska V., Obertova J., Palacka P. (2016). Prognostic value of serum carbonic anhydrase IX in testicular germ cell tumor patients. Oncol. Lett..

[30] Ouyang J., Qin G., Liu Z., Jian X., Shi T., Xie L. (2022). ToPP: Tumor online prognostic analysis platform for prognostic feature selection and clinical patient subgroup selection. iScience.

[31] Jézéquel P., Gouraud W., Ben Azzouz F., Guérin-Charbonnel C., Juin P.P., Lasla H., Campone M. (2021). bc-GenExMiner 4.5: New mining module computes breast cancer differential gene expression analyses. Database.

[32] Amat S., Penault-Llorca F., Cure H., Le Bouedëc G., Achard J.-L., Van Praagh I., Feillel V., Mouret-Reynier M.-A., Dauplat J., Chollet P. (2002). Scarff-Bloom-Richardson (SBR) grading: A pleiotropic marker of chemosensitivity in invasive ductal breast carcinomas treated by neoadjuvant chemotherapy. Int. J. Oncol..

[33] D’Eredita’ G., Giardina C., Martellotta M., Natale T., Ferrarese F. (2001). Prognostic factors in breast cancer: The predictive value of the Nottingham Prognostic Index in patients with a long-term follow-up that were treated in a single institution. Eur. J. Cancer.

[34] Fong Y., Evans J., Brook D., Kenkre J., Jarvis P., Gower-Thomas K. (2015). The Nottingham Prognostic Index: Five- and ten-year data for all-cause Survival within a Screened Population. Ann. R. Coll. Surg. Engl..

[35] Hussain S.A., Ganesan R., Reynolds G., Gross L., Stevens A., Pastorek J., Murray P.G., Perunovic B., Anwar M.S., Billingham L. (2007). Hypoxia-regulated carbonic anhydrase IX expression is associated with poor survival in patients with invasive breast cancer. Br. J. Cancer.

[36] Generali D., Fox S.B., Berruti A., Brizzi M.P., Campo L., Bonardi S., Wigfield S.M., Bruzzi P., Bersiga A., Allevi G. (2006). Role of carbonic anhydrase IX expression in prediction of the efficacy and outcome of primary epirubicin/tamoxifen therapy for breast cancer. Endocr. Relat. Cancer.

[37] Tomes L., Emberley E., Niu Y., Troup S., Pastorek J., Strange K., Harris A., Watson P.H. (2003). Necrosis and hypoxia in invasive breast carcinoma. Breast Cancer Res. Treat..

[38] Neumeister V.M., Sullivan C.A., Lindner R., Lezon-Geyda K., Li J., Zavada J., Martel M., Glazer P.M., Tuck D.P., Rimm D.L. (2012). Hypoxia-induced protein CAIX is associated with somatic loss of BRCA1 protein and pathway activity in triple negative breast cancer. Breast Cancer Res. Treat..

[39] Krieg A., Mwahech-Fauceglia P., Lim J., Pejovic T. (2017). Co-expression of the Hypoxic Marker Carbonic Anhydrase 9 (CA-IX) with Breast Cancer Associated 1 (BRCA1) is associated with faster recurrence in High Grade Serous Adenocarcinoma. Gynecol. Oncol..

[40] Rafajová M., Zatovicová M., Kettmann R., Pastorek J., Pastoreková S. (2004). Induction by hypoxia combined with low glucose or low bicarbonate and high posttranslational stability upon reoxygenation contribute to carbonic anhydrase IX expression in cancer cells. Int. J. Oncol..

[41] Olive P.L., Aquino-Parsons C., MacPhail S.H., Liao S.Y., Raleigh J.A., Lerman M.I., Stanbridge E.J. (2001). Carbonic anhydrase 9 as an endogenous marker for hypoxic cells in cervical cancer. Cancer Res..

[42] Barathova M., Takacova M., Holotnakova T., Gibadulinova A., Ohradanova A., Zatovicova M., Hulikova A., Kopacek J., Parkkila S., Supuran C.T. (2008). Alternative splicing variant of the hypoxia marker carbonic anhydrase IX expressed independently of hypoxia and tumour phenotype. Br. J. Cancer.

[43] Švastová E., Žilka N., Zat’ovičová M., Gibadulinová A., Čiampor F., Pastorek J., Pastoreková S. (2003). Carbonic anhydrase IX reduces E-cadherin-mediated adhesion of MDCK cells via interaction with β-catenin. Exp. Cell Res..

[44] Zatovicova M., Jelenska L., Hulikova A., Csaderova L., Ditte Z., Ditte P., Goliasova T., Pastorek J., Pastorekova S. (2010). Carbonic Anhydrase IX as an Anticancer Therapy Target: Preclinical Evaluation of Internalizing Monoclonal Antibody Directed to Catalytic Domain. Curr. Pharm. Des..

[45] Bourseau-Guilmain E., Menard J.A., Lindqvist E., Indira Chandran V., Christianson H.C., Cerezo Magaña M., Lidfeldt J., Marko-Varga G., Welinder C., Belting M. (2016). Hypoxia regulates global membrane protein endocytosis through caveolin-1 in cancer cells. Nat. Commun..

[46] Zaťovičová M., Pastoreková S. (2013). Modulation of cell surface density of carbonic anhydrase IX by shedding of the ectodomain and endocytosis. Acta Virol..

[47] Famta P., Shah S., Khatri D.K., Guru S.K., Singh S.B., Srivastava S. (2022). Enigmatic role of exosomes in breast cancer progression and therapy. Life Sci..

[48] Buanne P., Renzone G., Monteleone F., Vitale M., Monti S.M., Sandomenico A., Garbi C., Montanaro D., Accardo M., Troncone G. (2013). Characterization of Carbonic Anhydrase IX Interactome Reveals Proteins Assisting Its Nuclear Localization in Hypoxic Cells. J. Proteome Res..

[49] Barsoum I.B., Hamilton T.K., Li X., Cotechini T., Miles E.A., Siemens D.R., Graham C.H. (2011). Hypoxia Induces Escape from Innate Immunity in Cancer Cells via Increased Expression of ADAM10: Role of Nitric Oxide. Cancer Res..

[50] Rzymski T., Petry A., Kračun D., Rieß F., Pike L., Harris A.L., Görlach A. (2012). The unfolded protein response controls induction and activation of ADAM17/TACE by severe hypoxia and ER stress. Oncogene.

[51] McGowan P.M., McKiernan E., Bolster F., Ryan B.M., Hill A.D.K., McDermott E.W., Evoy D., O’Higgins N., Crown J., Duffy M.J. (2008). ADAM-17 predicts adverse outcome in patients with breast cancer. Ann. Oncol..

[52] Mullooly M., McGowan P.M., Kennedy S.A., Madden S.F., Crown J., O’ Donovan N., Duffy M.J. (2015). ADAM10: A new player in breast cancer progression?. Br. J. Cancer.

[53] Jackson H.W., Defamie V., Waterhouse P., Khokha R. (2017). TIMPs: Versatile extracellular regulators in cancer. Nat. Rev. Cancer.

[54] Cheng G., Fan X., Hao M., Wang J., Zhou X., Sun X. (2016). Higher levels of TIMP-1 expression are associated with a poor prognosis in triple-negative breast cancer. Mol. Cancer.

[55] Kajanova I., Zatovicova M., Jelenska L., Sedlakova O., Barathova M., Csaderova L., Debreova M., Lukacikova L., Grossmannova K., Labudova M. (2020). Impairment of carbonic anhydrase IX ectodomain cleavage reinforces tumorigenic and metastatic phenotype of cancer cells. Br. J. Cancer.

[56] Della Rocca Y., Fonticoli L., Rajan T.S., Trubiani O., Caputi S., Diomede F., Pizzicannella J., Marconi G.D. (2022). Hypoxia: Molecular pathophysiological mechanisms in human diseases. J. Physiol. Biochem..

[57] Tal R. (2012). The Role of Hypoxia and Hypoxia-Inducible Factor-1Alpha in Preeclampsia Pathogenesis. Biol. Reprod..

[58] Galbiati S., Gabellini D., Ambrosi A., Soriani N., Pasi F., Locatelli M., Lucianò R., Candiani M., Valsecchi L., Zerbini G. (2023). Early increase in circulating carbonic anhydrase IX: A potential new predictive biomarker of preeclampsia. Front. Mol. Biosci..

[59] Grossmannova K., Barathova M., Belvoncikova P., Lauko V., Csaderova L., Tomka J., Dulka T., Pastorek J., Madaric J. (2022). Hypoxia Marker Carbonic Anhydrase IX Is Present in Abdominal Aortic Aneurysm Tissue and Plasma. Int. J. Mol. Sci..

[60] Geçkil A.A., Kıran T.R., Berber N.K., Otlu Ö., Erdem M., İn E. (2022). Carbonic Anhydrase IX as a Marker of Disease Severity in Obstructive Sleep Apnea. Medicina.

[61] Merkx R.I.J., Lobeek D., Konijnenberg M., Jiménez-Franco L.D., Kluge A., Oosterwijk E., Mulders P.F.A., Rijpkema M. (2021). Phase I study to assess safety, biodistribution and radiation dosimetry for 89Zr-girentuximab in patients with renal cell carcinoma. Eur. J. Nucl. Med. Mol. Imaging.

[62] Verhoeff S.R., van Es S.C., Boon E., van Helden E., Angus L., Elias S.G., Oosting S.F., Aarntzen E.H., Brouwers A.H., Kwee T.C. (2019). Lesion detection by [89Zr]Zr-DFO-girentuximab and [18F]FDG-PET/CT in patients with newly diagnosed metastatic renal cell carcinoma. Eur. J. Nucl. Med. Mol. Imaging.

[63] Zatovicova M., Kajanova I., Barathova M., Takacova M., Labudova M., Csaderova L., Jelenska L., Svastova E., Pastorekova S., Harris A.L. (2022). Novel humanized monoclonal antibodies for targeting hypoxic human tumors via two distinct extracellular domains of carbonic anhydrase IX. Cancer Metab..

[64] Schütze D., Milde-Langosch K., Witzel I., Rody A., Karn T., Schmidt M., Choschzick M., Jänicke F., Müller V. (2013). Relevance of cellular and serum carbonic anhydrase IX in primary breast cancer. J. Cancer Res. Clin. Oncol..

[65] Gradishar W.J., Moran M.S., Abraham J., Aft R., Agnese D., Allison K.H., Anderson B., Burstein H.J., Chew H., Dang C. (2022). Breast Cancer, Version 3.2022, NCCN Clinical Practice Guidelines in Oncology. J. Natl. Compr. Canc. Netw..

[66] Ellis I.O., Rakha E.A., Tse G.M., Tan P.H. (2023). An international unified approach to reporting and grading invasive breast cancer. An overview of the International Collaboration on Cancer Reporting (ICCR) initiative. Histopathology.

